# Structural and functional analysis of the newt lymphatic system

**DOI:** 10.1038/s41598-023-34169-w

**Published:** 2023-04-27

**Authors:** Chihena H. Banda, Makoto Shiraishi, Kohei Mitsui, Yoshimoto Okada, Kanako Danno, Ryohei Ishiura, Kaho Maemura, Chikafumi Chiba, Akira Mizoguchi, Kyoko Imanaka-Yoshida, Kazuaki Maruyama, Mitsunaga Narushima

**Affiliations:** 1 Department of Plastic and Reconstructive Surgery, Graduate School of Medicine, Mie University, 2-174 Edobashi, Tsu, Mie Prefecture 514-8507 Japan; 2 Faculty of Life and Environmental Sciences, University of Tsukuba, 1-1-1 Tennodai, Tsukuba, Ibaraki Prefecture 305-8571 Japan; 3 Department of Personalized Cancer Immunotherapy, Graduate School of Medicine, Mie University, 2-174 Edobashi, Tsu, Mie Prefecture 514-8507 Japan; 4 Department of Pathology and Matrix Biology, Graduate School of Medicine, Mie University, 2-174 Edobashi, Tsu, Mie Prefecture 514-8507 Japan

**Keywords:** Imaging, Regeneration, Animal physiology, Imaging the immune system, Lymphatic system, Lymphangiogenesis

## Abstract

Regeneration competent vertebrates such as newts and salamanders possess a weakened adaptive immune system characterized by multiple connections between the lymphatic system and the blood vascular system called lymphatic hearts. The role of lymphatic vasculature and these lymphaticovenous connections in regeneration is unknown. We used in-vivo near-infrared lymphangiography, ultra-high frequency ultrasonography, micro-CT lymphangiography, and histological serial section 3-dimentional computer reconstruction to evaluate the lymphatic territories of *Cynops pyrrhogaster*. We used our model and supermicrosurgery to show that lymphatic hearts are not essential for lymphatic circulation and limb regeneration. Instead, newts possess a novel intraosseous network of lymphatics inside the bone expressing VEGFR-3, LYVE-1 and CD-31. However, we were unable to show Prox-1 expression by these vessels. We demonstrate that adult newt bone marrow functions as both a lymphatic drainage organ and fat reservoir. This study reveals the fundamental anatomical differences between the immune system of urodeles and mammals and provides a model for investigating lymphatics and regeneration.

## Introduction

The lymphatics system consists of a network of lymphatic vessels, lymphoid tissue and lymphoid organs that form an important part of the immune system in vertebrates. Lymphatics begin as blind ended vessels in tissue and form an extensive network throughout the body but have not yet been identified in the epidermis, cartilage, nor in healthy bone. All lymphatics ultimately drain into blood circulation through direct lymphatic to vein (lymphaticovenous) connections.

The lymphatic system functions in regulating the surrounding environment of cells within tissues by controlling the availability of fluid, macromolecules, and white blood cells, forming an important bridge between the cells, extracellular matrix, and the blood vascular system. Asides their traditionally recognized role in extracellular fluid transportation, recent studies have shown lymphatics also play an essential role in wound healing, immunity, fat metabolism, cancer metastasis, cardiovascular and metabolic diseases^1–6^. Failure of lymphatic vasculature regeneration following injury results in lymphedema, a disfiguring disease characterized by progressive limbs swelling, fat deposition and repeated cellulitis infections that affects over 200 million people worldwide and has no known cure^7^.

Despite the growing literature on the importance of lymphatics in wound healing and disease, the role of the lymphatic system in regeneration remains poorly studied. A major reason for this is the lack of knowledge of the lymphatic system in urodele amphibians (newts and salamanders) widely considered the best models for vertebrate regeneration due to their unmatched regenerative ability^8^. Much of the current knowledge on urodele lymphatics is based on studies carried out over a century ago and on assumptions made from anuran (frogs and toads) research^9–11^.

The urodele’s lymphatic system is characterized by the presence of 8 to 23 pairs of lymphatic hearts (LH) located at the lymphaticovenous connections along the length of the body that function to pump lymph into veins and prevent backflow of blood into lymphatics^8,10,12^. LHs are not unique to newts. Anurans have 2 to 5 pairs, while reptiles and birds possess one pair that disappears in most birds in the early post embryonic period^10^. Mammals on the other hand, do not possess LHs, instead, the initial veno-lymphatic plexus that forms during jugular lymph sac development has been described as the vestigial homologue of the LH^13,14^. Therefore, post embryonic mammals have only 1 pair of lymphaticovenous connections common to all tetrapods located around the junction of the thoracic ducts and subclavian veins. These significant anatomical differences in vertebrate lymphatic circulation have correspondingly significant implications on lymphatic system function.

The decrease in lymphaticovenous connections remarkably correlates with decreased regeneration ability in vertebrates (Supplementary Fig. i). Evidence of this relationship is vividly conspicuous in the life cycle of anurans. As larvae tadpoles they share an identical lymphatic structure as that of urodeles with 8 or more pairs of LHs giving a total of at least 18 lymphaticovenous connections (including the thoracic duct to subclavian vein connections). During this phase of life, tadpoles, like urodeles possess the ability to regenerate tails, limbs, and the lens of the eye^15–17^. They then undergo an ontogenic decline, transitioning to a regeneration-incompetent state as adults with a corresponding decline in the number of LHs to 2 to 5 pairs^15–17^. Studies in the South African clawed frog *Xenopus laevis* have shown that tadpoles up to stage 51 to 52 are able to regenerate a complete hindlimb with all 5 digits following amputation^17,18^. This progressively declines to 3.7 digits on average at stage 53, and 2.5 at stage 55, accompanied by progressive loss of sonic hedgehog expression in the regenerating tissue^17–19^. Following metamorphosis, hindlimb regeneration is limited to formation of a cartilaginous spike lacking any digits or muscle^18,20^. The etiology of this progressive decline in regenerative ability in anurans and in higher vertebrates such as mammals remains unknown, with the leading hypothesis attributing this decline to the differences in the immune system response to injury^20–24^. Furthermore, whether the presence of these multiple lymphaticovenous connections facilitates regeneration equally remains unknown.

In this study we defined the functional lymphatic anatomy of the Japanese fire belly newt *Cynops pyrrhogaster*. We used our model to show that in contrast to anurans, LH lymphaticovenous connections are not essential for lymphatic circulation and are not essential for regeneration. Instead, newts possess a novel intraosseous network of lymphatics expressing vascular endothelial growth factor receptor 3 (VEGFR-3), lymphatic vessel endothelial hyaluronan receptor 1 (LYVE-1) and CD-31 that connects the superficial and deep lymphatic networks and maintains lymphatic circulation even after excision of all the LHs. This is the first report of lymphatics in healthy bone and provides insight on the fundamental anatomical differences in the immune system of the highly regenerative urodeles and mammals. This study also provides a model for investigating the role of lymphatic vasculature in regeneration and the regeneration of lymphatics in urodeles.

Notably, there is considerable variation in the anatomical nomenclature used in urodeles. This is largely due to the paucity of literature on the vascular anatomy of urodeles and historically conflicting findings by early researchers that have persisted. The landmark monograph by Francis published in 1934, *The Anatomy of the Salamander*, includes the most detailed anatomical description of adult salamander vasculature to date^9^. Therefore, the nomenclature used in this study is in line with this monograph^9^.

## Results

### Newt lymphatic circulation is organized into defined lymphatic territories

We first evaluated the lymphatic drainage territories in-vivo using indocyanine green (ICG) near-infrared fluoroscopy (NIRF) lymphangiography complimented with ultra-high frequency ultrasonography (UHFUS) and micro-computer tomography (micro-CT) contrast lymphangiography. NIRF has high resolution but poor tissue penetration depth while UHFUS (40–70 MHz) is useful for measurement of size and pulsation rates but has relatively poor resolution and low penetration depth. Micro-CT on the other hand allows visualization of deep lymphatic networks to complement NIRF and UHFUS. Collecting lymphatic vessels were defined functionally as the larger lymphatics gathering distant flow.

**Figure 1:**
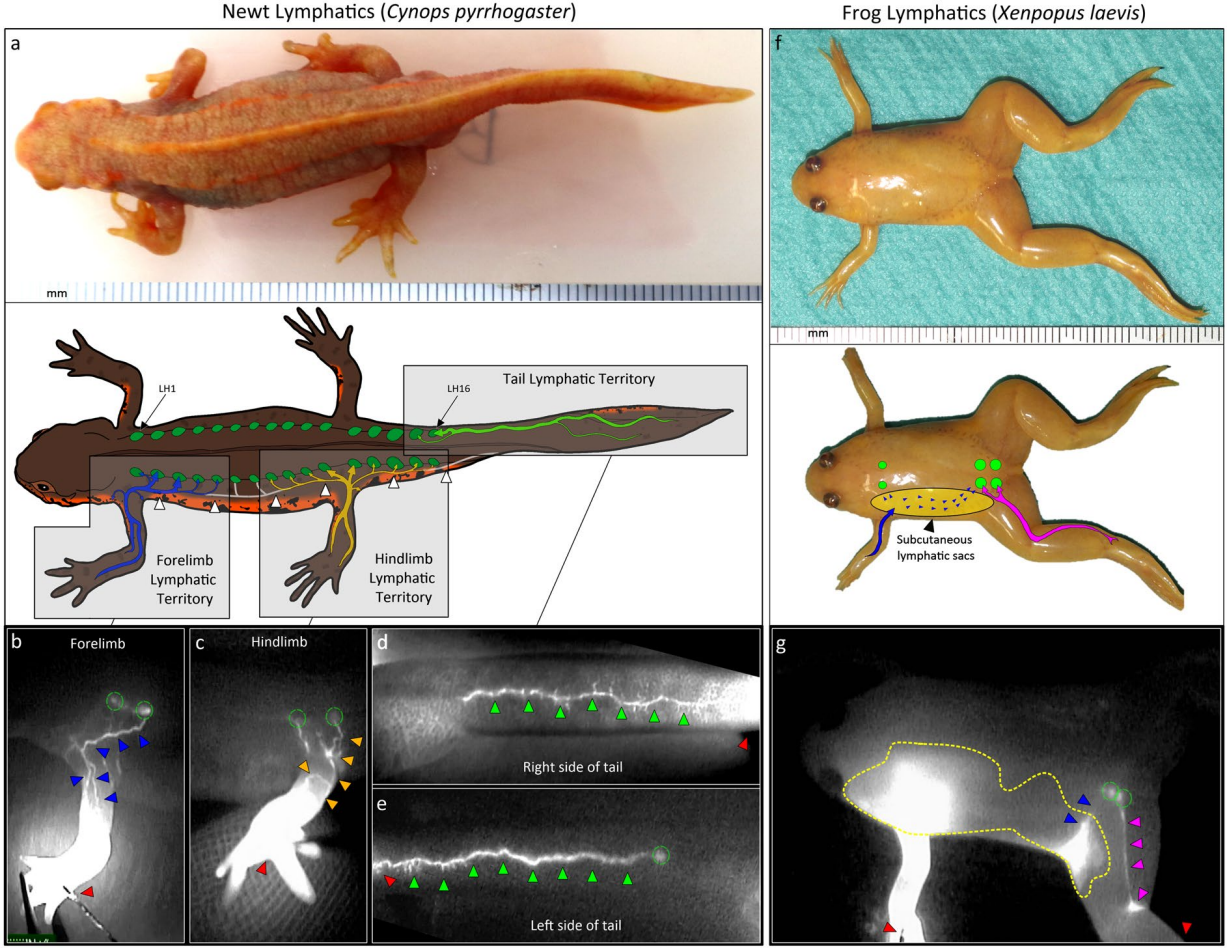
Lymphatic territories mapped using in-vivo indocyanine green near infrared fluoroscopic lymphangiography. (**a**) Diagram showing the lymphatic flow territories of the newt forelimb (blue), hindlimb (yellow) and tail (green) mapped using indocyanine green (ICG) near infra-red fluoroscopy (NIRF). The trunci lymphatici longitudinales lateralis (TLyLL) (white arrowheads) run along the body interconnecting all the lymphatic hearts (LH) (green circles). (**b**) Forelimb lymphatics (blue arrowheads) run anteriorly to a dense axilla network and primarily drained into LH2 and 3 (green circles) then LH1, 3 and 5. Collecting lymphatics from the axilla crossed the mid-line on the ventral surface to join the collectors of the head region and the contralateral forelimb. The ICG injection sites are shown with red arrowheads. (**c**) Hindlimb lymphatics (yellow arrowheads) run posteriorly and flowed primarily to LH11 and LH12 (green circles) then to LH8, 9, 10, 13, 14 and 15. The hindlimb lymphatics connected to a dense network around the cloaca. Lymphatics on the right side (**d**) and left side (**e**) of the tail (green arrowheads) were drained by respective ipsilateral lymphatic vessels with the main collectors on the left and right running a slightly different course anteriorly and proceeded to form the TLyLL on each side. Lymphatic fluid from the tail drained into LH16 (green circle) and later LH15 through the TLyLL. (**f**) Diagram showing the lymphatic flow territories of the frog forelimb (blue) and hindlimb (magenta) mapped using ICG NIRF. The large subcutaneous lymphatic sacs (yellow) connecting the limb lymphatics and the LH (green circles). (**g**) The frog hindlimb was drained mainly by dorsal collectors (magenta arrowheads) to the posterior LHs (green circles) and some fluid flowed to the large subcutaneous lymphatic sacs (yellow outline). Forelimb lymphatics flowed rapidly into the lymphatic sacs obscuring the deeper lymphatic vasculature before flowing to the posterior LHs (blue arrowheads). The ICG injection sites are shown with red arrowheads. No subcutaneous lymphatic sacs were seen in newts.

We identified 16 pairs of lateral LHs located on the back of the newt from the scapulae to the base of the tail and numbered these LH1 to LH16 respectively. ICG rapidly flowed to the LHs 2–5 min after injection and peaked in 10–15 min. The limbs and the tail were consistently drained by well-defined collecting lymphatic vessels into specific LHs (Fig. 1a). In the forelimb, lymphatics run on the dorsal aspect of the limb posteriorly to an axilla network then drained primarily into LH2 and LH3, then to LH1, LH4 and LH5 through truncus lymphaticus logitudinalis lateralis (TLyLL) connections. Lymphatic collectors on the ventral surface connected the axilla network to collectors of the head and contralateral forelimb (Fig. 1b). Similarly, in the hindlimb, 2 main collecting lymphatics on the dorsum run posteriorly to the inferior pelvis then drained into LH11 and LH12 and later to LH8, LH9, LH10, LH13, LH14, LH15 and occasionally LH16 through TLyLL connections (Fig. 1c). Collecting vessels also run anteriorly, ventrally crossing the midline and connecting to a network around the cloaca and to collectors of the contralateral hindlimb. The tail was drained by 2 collectors running along the lateral ridge on the left and right sides that continued anteriorly to form the TLyLL and drained into LH16 and later LH15 (Fig. 1d,e).

In *Xenopus*, hindlimb drainage was mainly through dorsal collectors to the posterior LHs and sacs (Fig. 1f,g). In the forelimb, ICG rapidly collected in large subcutaneous lymphatic sacs obscuring smaller vasculature. Fluid from the lymphatic sacs then flowed to the posterior LHs. We found no such lymphatic sacs in newts. Instead, excess dye collected in extravascular spaces especially around the pelvis and axilla subcutaneous tissue and along nerve sheaths that were not lined by endothelium, confirmed by the absence of endothelial markers VEGFR-3, LYVE-1 and CD-31 (Supplementary Figs. ii and iii). No fluid was seen collecting in these spaces when small amounts of contrast dye was injected suggesting lymphatic flow in these extravascular pathways has a limited role in physiological circulation. No deaths or changes in movement or feeding behavior after ICG administration were observed in animals followed-up for up to 4 years.

### Newt lymphatic hearts have high drainage capacity

The LH rate at rest varied from 60 to 122 beats per min (bpm) interspaced with periods of asystole and increased to 72–156 bpm after injection of 50 μL dye. Each LH beat independently with an irregular rhythm and there was no synchronicity between adjacent ipsilateral or contralateral LHs. Pulsation rates were generally similar in all the LHs. Dye injection into one region caused simultaneous increase in the rate of all the LHs, including those not sharing the same drainage territory with no dye collection (data not shown) suggesting centralized autonomic nervous system control.

**Figure 2:**
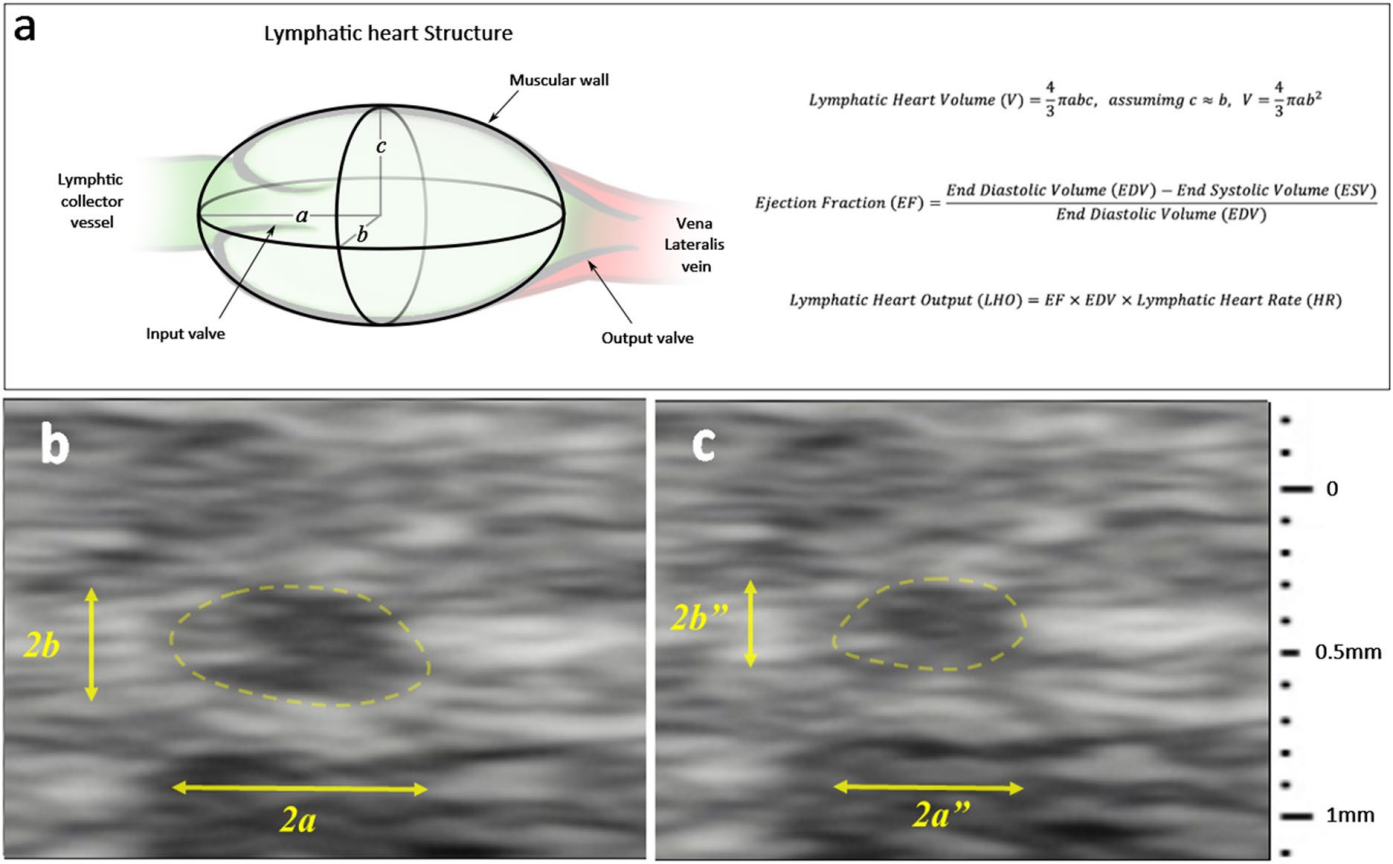
Ultra-high frequency ultrasound-based calculation of the newt lymphatic heart cardiac output. (**a**) The lymphatic heart (LH) structure consists of a single muscular chamber receiving lymphatic fluid from the lymphatic collectors and pumping this fluid into the vena lateralis vein. The LH has one input valve and one output valve that prevent backflow of blood. Computer 3D volume reconstruction showed the newt lymphatic heart shape conformed best to an ellipsoid-like shape with a slightly pointed output end and flattened input end, therefore, the ejection fraction (EF) was calculated using an ellipsoid mathematical model based on 2D ultra-high frequency ultrasound measurement of the LH end diastolic volume (EDV) (**b**) and end systolic volume (**c**), and the LH rate. The mean EF was 60.40% (SD 12.94), EDV 0.026 mm^3^ (SD 0.008) and the mean lymphatic heart rate was 117.04 bpm (SD 23.12), giving a LH cardiac output (LHO) of 1.838 mm^3^/min for each LH and a combined total of 58.816 mm^3^/min for all 16 pairs.

An ellipsoid mathematical model (Fig. 2a) was used to estimate the ejection fraction (EF) on UHFUS and to calculate the LH cardiac output (LHO) (Fig. 2b,c). The mean EF was 60.40%, EDV 0.026 mm^3^, LH rate 117.04 bpm, LHO 1.838 mm^3^/min per LH and the combined total for all 16 pairs was 58.816 mm^3^/min. Thus, newts are remarkably capable of pumping a volume of lymphatic fluid equivalent of their 5 to 6 g body mass every 1 to 2 h into peripheral venous circulation at a rate of 10 ml.Kg^−1^.min^−1^.

### Newts possess a novel intraosseous lymphatic network inside bone marrow

**Figure 3:**
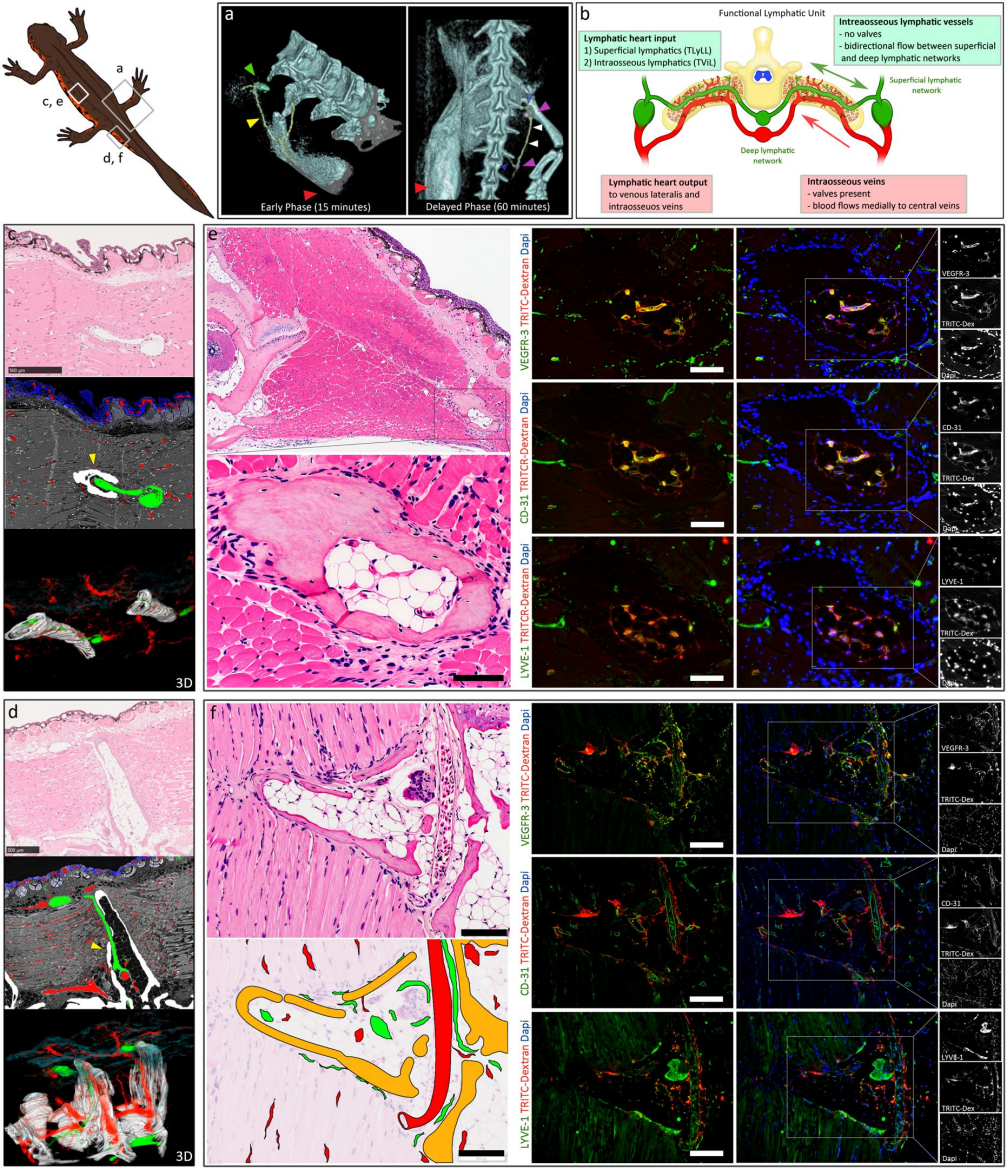
Newt microcirculation and the intraosseous bone marrow lymphatic network. (**a**) Micro-CT Lymphangiography showed movement of contrast injected in the left hindlimb (red arrowhead) flowed through collectors (yellow arrowheads) to the lymphatic hearts (LH) (green arrowhead) and into the vertebral bone to the contralateral collectors (blue arrowhead), contralateral LHs (magenta arrowheads) and the trunci lymphatici longitudinales lateralis (TLyLL) (white arrowheads). (**b**) The functional lymphatic unit repeated throughout the trunk of the newt. The LH input from the superficial lymphatic network via the TLyLL and the transverse vertebral intraosseous lymphatics (TvIL) connecting the deep lymphatic network to the superficial lymphatic network and LHs on both sides of the body. (**c**) and (**d**) Serial section computer 3D volume reconstruction showing the lymphatic vessels (green) and blood vessels (red). The TvIL (yellow arrowheads) are shown flowing from the bone to the LHs. The anterior LHs of the trunk (**c**) were more closely attached to the adjacent rib bone while in the tail (**d**) they were separated from the bones by skeletal muscle. (**e**) and (**f**) Immunohistochemistry showing intraosseous lymphatic vessels and blood vessels in the bone marrow of the transverse process of an abdominal rib (Scale bars = 100 μm) (**e**) and the tail vertebra (Scale bars = 200 μm) (**f**). Lymphatic endothelial cells were identified by high rhodamine-dextran uptake and VEGFR-3 + , LYVE-1 + , CD-31 + staining while blood vessel endothelial cells were CD-31 + , LYVE-1 ± , VEGFR-3- and low rhodamine-dextran uptake.

To evaluate the deep lymphatic networks, we performed micro-CT lymphangiography which showed flow of lymphatic fluid across the midline to the contralateral side of the body through the ribs, vertebra and deep vertebral lymphatics (Fig. 3a). We proceeded to evaluate newt microcirculation to confirm the location of these alternative lymphatic pathways using serial sectioning with computer 3-Dimentional (3D) volume reconstruction supported with immunohistochemistry and transmission electron microscopy (TEM). To ensure clearance of red blood cells, prevent collapse of lymphatic vessel lumens and for enhanced fixation we prepared the tissue for vascular 3D volume reconstruction by fixation with perfusion via the lymphatic system. Fixation by trans-lymphatic perfusion utilized the rapid lymphatic circulation, multiple anatomical lymphaticovenous connections and the sparsity of venous valves that allow bi-directional flow to capillary beds to adequately fix the newt and to prevent collapse of lymphatic vessels. Urodeles have 4 pairs of large collecting lymphatic vessels that run longitudinally along the trunk, the TLyLL which runs along the LHs and directly supplies them, trunci lymphatici longitudinales parabdominales (TLyLPab) and trunci lymphatici longitudinales parepigastrici (TLyLPe) which run on the lateral and ventral subcutaneous layers of the trunk wall respectively, and trunci lymphatici longitudinales subvertebrales (TLyLSv) which are the largest of the four and run deep in the trunk immediately ventral to the vertebral column^9^.

The lymphatic network was functionally organized into a superficial dermal and subdermal network, and a deep visceral network with a few small vessels seen traversing the muscle layer mainly in the intermuscular septa connecting the two (Supplementary Figs. iv and v). The superficial network included a fine dermal lymphatic plexus closely associated with the mucus and poison glands of the skin and a subdermal plexus which contained the TLyLL, TLyLPab, TLyLPe and the body segmental collectors. The deep lymphatic network included the TLyLSv, vertebral plexus of lymphatics and the deep vessels running alongside the major blood vessels and nerves in the intermuscular spaces to join the deep intraabdominal and intrathoracic visceral lymphatics eventually draining into the major veins to the blood heart. No lymphatics were seen in the epidermis. Similarly, no blood vessels were observed in the epidermis. Instead, the blood vessels formed an extremely rich dense sub-epidermal plexus. The muscle also possessed an extensive blood vessel network connecting the superficial and deep blood vessels. The venous lateralis (VL) receiving LH output run in the subdermal layer alongside the TLyLL.

We found that the LH had 2 main inputs, the TLyLL collecting superficial lymphatics, and a novel intraosseous lymphatic network in bone connecting to the TLyLSv deep lymphatics (Fig. 3b). The intraosseous lymphatic network consisted of one main collecting lymphatic vessel we termed the transverse vertebral intraosseous lymphatic (TvIL) vessel inside the vertebral transverse processes and ribs accompanied by 1 to 2 veins connecting the VL LH output to the sub-vertebral venous plexus, we termed these the transverse vertebral intraosseous veins (TvIV) (Fig. 3c,d, Videos 1 and 2). Both the TvIL and the TvIV had small branches and tributaries forming a network inside the bone marrow connecting to the periosteum and the surrounding muscle. The veins had markedly more tributaries and were characterized by the presence of multiple valves directing the flow of blood medially to the sub-vertebral venous plexus. Asides the 2 valves at the input and output ends of the LHs, no other valves were seen in the lymphatic vasculature, allowing for easy bidirectional fluid flow and rapid response to changes in lymph circulation.

**Figure 4:**
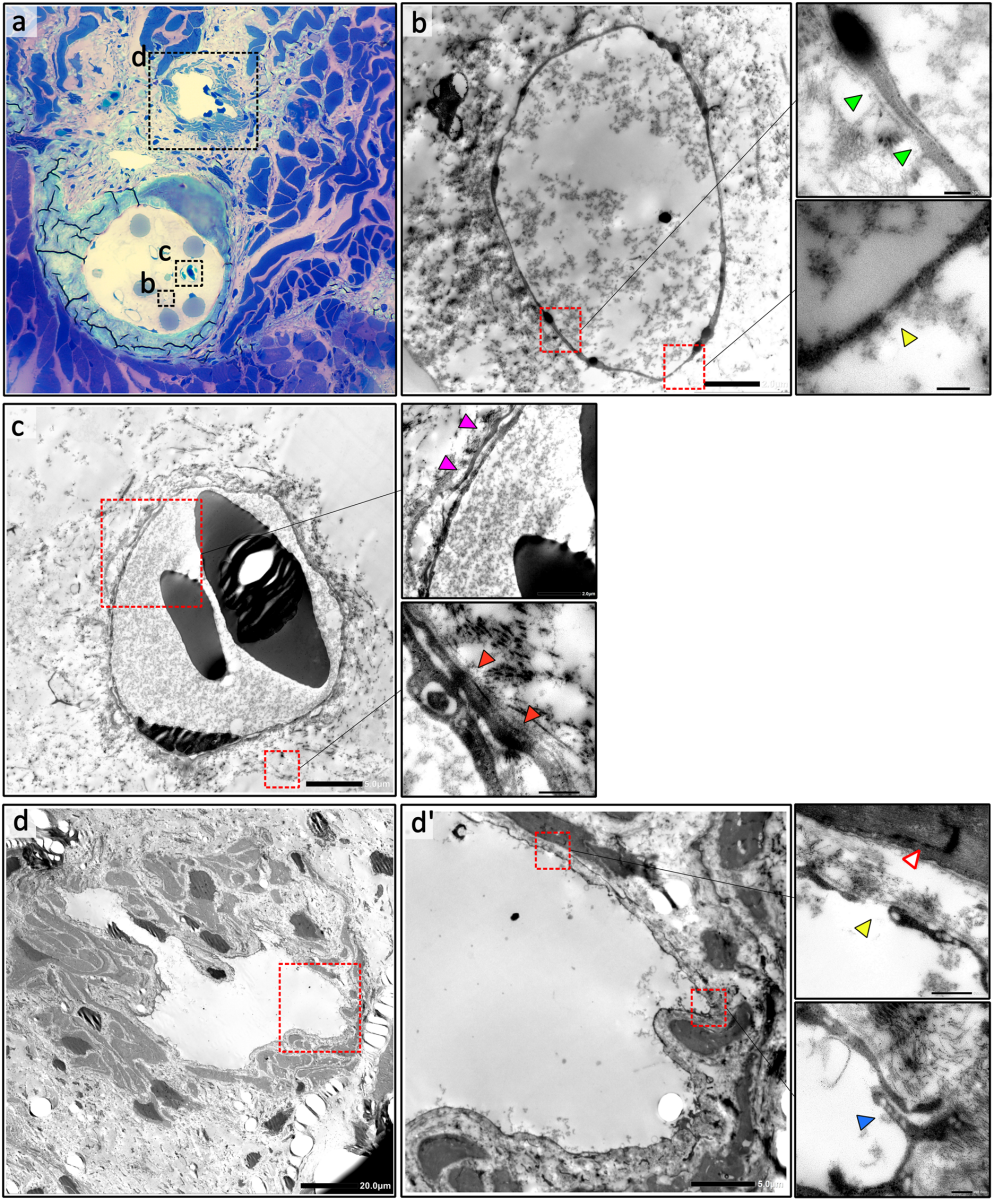
Ultra-structure of the intraosseous lymphatics and veins and the lymphatic heart. (**a**) Resin embedded toluidine blue stained scanning section of a newt abdominal rib showing the transverse vertebral intraosseous lymphatics (TvIL) (**b**) and the transverse vertebral intraosseous veins (TvIV) (**c**) inside the bone marrow, and the adjacent lymphatic heart (LH) (**d**). Transmission Electron Microscopy confirmed the TvIL ((**b**) Scale bar = 2 μm) and LH ((**d**) Scale bar = 20 μm) were lined by lymphatic endothelial cells (LEC) with the characteristic ultra-structural features of lymphatic endothelium including absence of pericytes, thin walls and highly attenuated LEC cytoplasm (green arrowheads (**b**)), with sparse tight junctions, few adherens junctions, occasional spaces between LECs (yellow arrowheads (**b and d'**)) and overlapping leaf-like endothelial cell junctions (blue arrowhead (**d'**)). The specialized LH muscle had characteristic features of both cardiac and skeletal muscle (white arrowhead (**d'**)). In contrast, the TvIV ((**c**) Scale bar = 5 μm) were lined by blood endothelial cells (BEC) had thicker walls, surrounded by pericytes (magenta arrowheads (**c**)) with several tight junctions and adherens junctions (red arrowheads (**c**)) and had multiple cytoplasm vesicles. Red blood cells were also seen in blood vessels.

The presence of a functional intraosseous lymphatic network was confirmed by the uptake of rhodamine-dextran dye in VEGFR-3 + , LYVE-1 + , CD-31 + vessels (Fig. 3e,f). TEM analysis (Fig. 4a–d) also confirmed the ultra-structure of lymphatic vessels inside the bone marrow and contrasted them to blood vessels^25^.

This structure consisting of the superficial lymphatic collectors, bringing lymph from the peripheral body segments to the LHs via the TLyLL, the TvIL transporting lymph between the deep lymphatics and LHs, and the LHs pumping lymph into the VL formed the basic functional lymphatic unit which was repeated along the entire trunk of the newt (Fig. 3b). In the anterior lymphatics draining the upper limb, chest and abdomen the TvIL run inside the ribs and was the larger, predominant input vessel of the LH while in the tail the superficial collectors draining the hindlimbs and tail were predominant. The anterior LHs (Video 1) were also more closely attached to the dorsal posterior aspect of the adjacent rib bone while in the tail region (Video 2) they were located slightly further from the bones separated by some skeletal muscle. Serial section analysis of all the bones of the forelimb and hindlimb, the ribs and vertebra of the neck, trunk and proximal tail, the shoulder girdle and pelvic girdle showed the newt bone marrow was highly vascular, hypocellular and extensively filled with adipose tissue (Supplementary Figs. vi and vii).

### Lymphatic heart excision causes edema and death in anurans but not in newts

**Figure 5:**
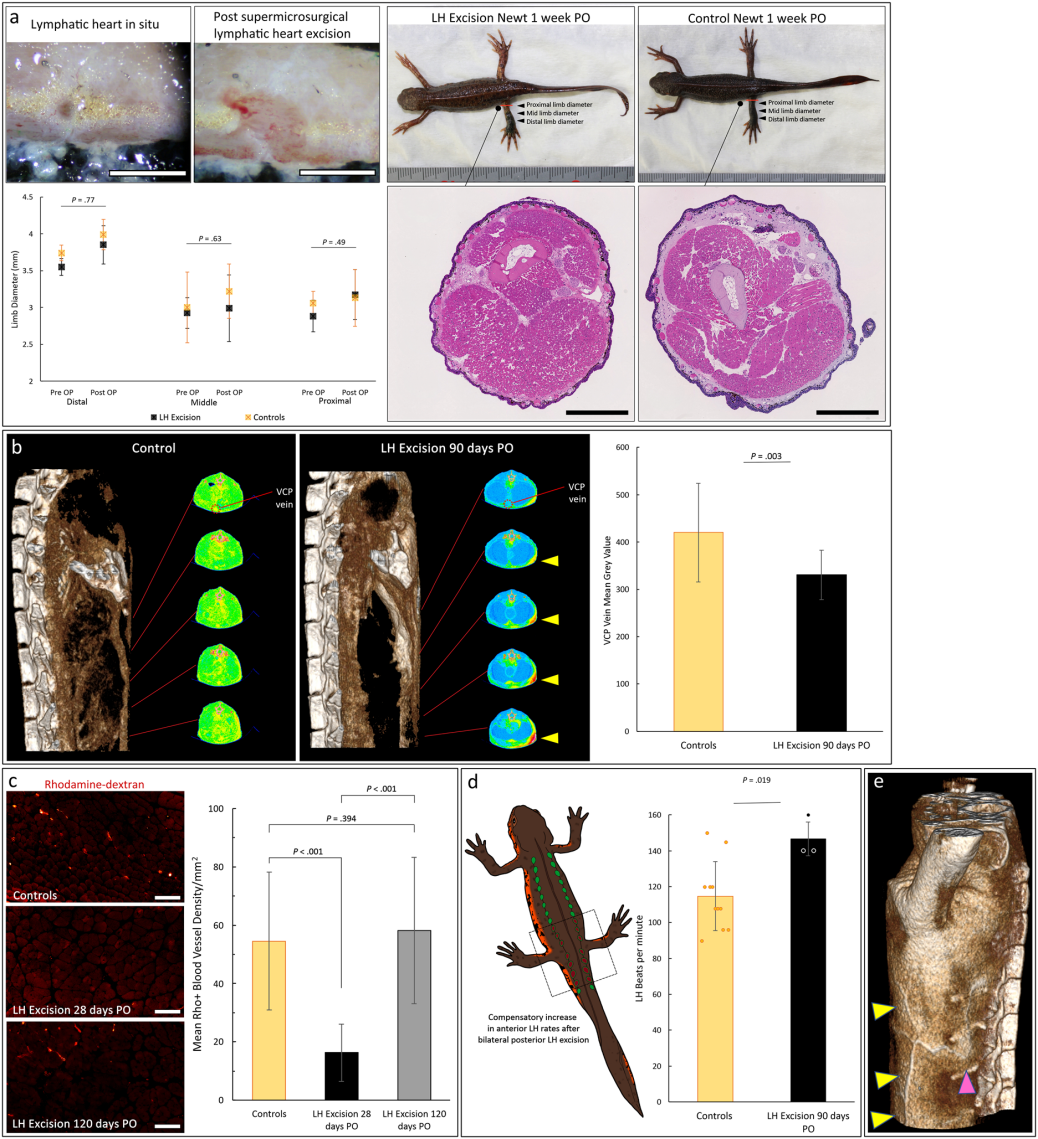
Physiological changes in lymphatic circulation following lymphatic heart excision. (**a**) Lymphatic hearts (LH) excision in newts did not cause edema, histological changes, nor death (Scale bars = 1 mm). There was no significant difference in the 1-week post operative (PO) limb diameters (proximal t(9) = 0.729, p = 0.485, middle t(9) = − 0.504, p = 0.627, distal t(9) = 0.297, p = 0.773) between LH excision newts and controls with good inter-rater reliability ICC = 0.893, 95% CI (0.825, 0.934). (**b**) Micro-CT lymphangiography showed reduced lymphatic contrast in the blood circulation in LH excision newts at 90 days PO, with Trunci lymphatici longitudinales parabdominales (TLyLPab) collateral flow seen (yellow arrowheads). Vena cava posterior (VCP) analysis showed LH excision newts had a significantly lower mean grey value (t(34) = − 3.156, p = 0.003) than controls. (**c**) Rhodamine-dextran fluorescence lymphangiography analysis of lymphatics to vein flow (Scale bars = 100 μm) also showed a significant decrease in the density of blood vessels receiving lymphatic fluid (t(188) = 13.057, p < 0.001) at 28 days post excision of all the LHs but this returned to normal at 120 days PO (t(161) = − 0.854, p = 0.394). (**d**) Ultra-high frequency ultrasonography showed a significant increase (t(12) = − 2.707, p = 0.019) in the LH rate in newts that had bilateral posterior LH excision. (**e**) Collateral flow maintained lymphatic circulation after excision of LHs. TLyLPab collateral is shown (yellow arrowheads) with connections to the TvIL (magenta arrowhead). These results confirmed that LH excision successfully terminated peripheral lymphatic to vein flow for 90 days following which normal lymphatic to vein flow was restored by 120 days PO. Data represent mean ± standard deviation (error bars).

LHs are essential for lymphatic circulation in anurans^13,26,27^. Experimental interruption of their function by anesthesia or ablation results in hemoconcentration, edema and inevitable death in frogs and toads in 2–4 days^13,26,27^. To evaluate differential LH function in newts and *Xenopus* we performed incremental supermicrosurgical LH excisions. Consistent with previous reports^13,26,27^, excision of the 2 pairs of posterior LHs in *Xenopus* resulted in edema, 18–29% post operative (PO) weight gain and death within 12 h in all the frogs despite the presence of intact anterior LHs (Supplementary Fig. viii). Interestingly, none of the newts developed edema, behavior changes or died in the 1 month to 1 year observation period following LH excision of regional or all 16 pairs of LHs (Fig. 5a).

### Lymphatic heart excision reduces peripheral lymphatic to vein flow

Quantitative assessment of lymphatic to vein flow was performed by quantitative micro-CT of the vena cava posterior (VCP) (Fig. 5b) and measuring the density of intramuscular blood vessels that had received rhodamine-dextran dye from lymphatics (Fig. 5c). The VCP is a major vein with a consistent course in newts. Unlike other major veins, we found that the VCP, much like the intramuscular blood vessels was not accompanied by multiple large lymphatics making them ideal for studying peripheral lymphatic to vein flow. LH excision newts had a significantly lower lymphatic to vein flow at 28 days PO (p < 0.001) and at 90 days (p = 0.003) that increased to normal values after 120 days (p = 0.394) confirming that LH removal effectively terminated the lymphaticovenous connections and reduced lymphatic to vein flow.

### Lymphatic heart compensation and collateral flow maintain lymphatic circulation

To determine why newts did not develop any symptoms of lymphatic dysfunction after LH excision we performed physiological circulatory assessment following excision of some or all the LHs. UHFUS showed the pumping rate of the remaining anterior LHs in newts that had excision of the posterior LHs was significantly higher than controls (Fig. 5d). This compensatory increased LH activity correspondingly increased the output of each LH by 20%, but the overall combined output of the remaining 9 pairs (41.454 mm^3^/min) was still lower than the combined output in normal newts. Micro-CT lymphangiography of LH excision newts showed the enlargement of collateral lymphatic vessels in the subdermal plane running longitudinally and transporting lymphatic fluid to the TvILs (Fig. 5b,e).

### Lymphatic heart regeneration occurs after regeneration of major blood vessels

**Figure 6:**
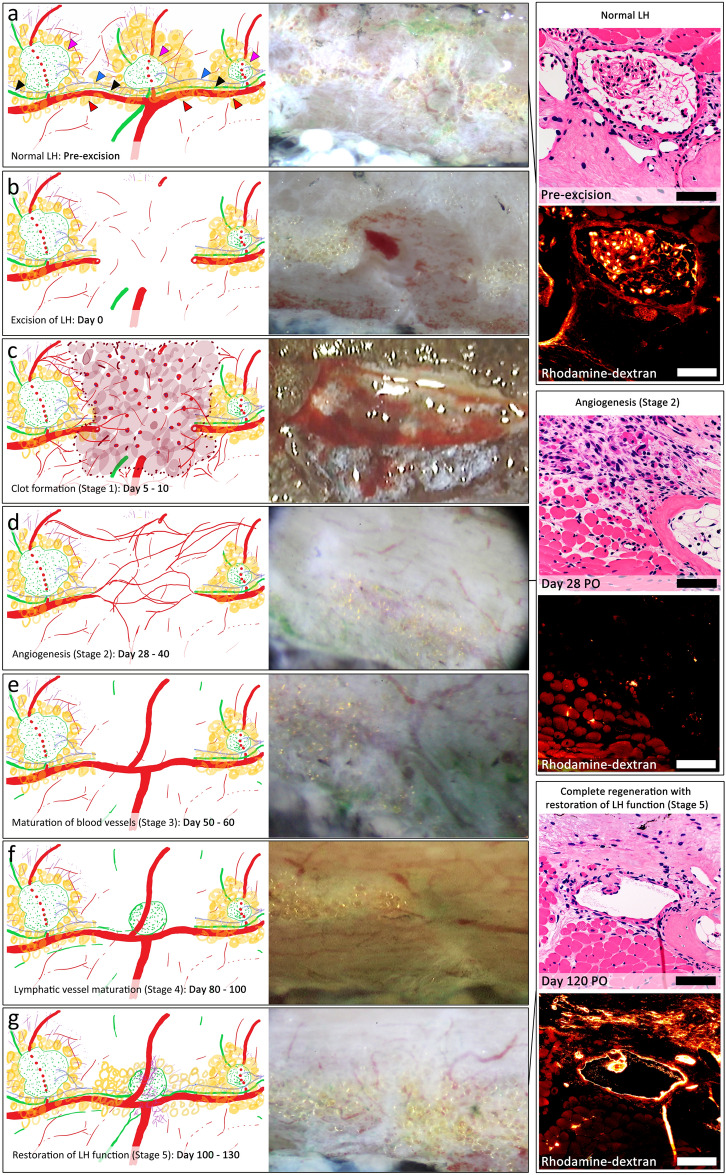
Morphological stages of lymphatic heart regeneration following complete excision. (**a**) Normal lymphatic heart (LH) (magenta arrowheads) with its supplying nerve (blue arrowheads), the trunci lymphatici longitudinales lateralis (TLyLL) (black arrowheads) and venous lateralis (VL) (red arrowheads). LH function was confirmed by collection of rhodamine-dextran (Scale bars = 100 μm). (**b**) The tissue defect immediately after LH excision. A segment of the TLyLL, VL and the supplying nerve, all of which are intimately associated with the LH were also resected. (**c**) Stage 1 Post Operative (PO) Day 5–10: Clot formation was followed by accumulation of a soft, fragile tissue mass beneath the wound epidermis. (**d**) Stage 2 PO Day 28–40: Angiogenesis of several small blood vessels interconnecting the cut ends of the VL and the other major blood vessels. No lymphangiogenesis seen at the injury site. Histology showed regenerating muscle and fibroblasts but no functional lymphatics with no rhodamine-dextran collection. (**e**) Stage 3 PO Day 50–60: Maturation of blood vessels restoring their original size and structure and restoration of VL continuity. Small lymphatic vessels were observed sprouting from the edges of the wound towards the injury zone. (**f**) Stage 4 PO Day 80–100: Lymphatic vessel maturation with active flow and collection of dye observed as a cystic mass in the region of the original LH, but no pulsations. (**g**) Stage 5 PO Day 100–130: Complete LH regeneration with pulsation. The regenerated LHs had fully functional input and output valves and uptake of rhodamine-dextran confirming they were fully functional.

**Figure 7:**
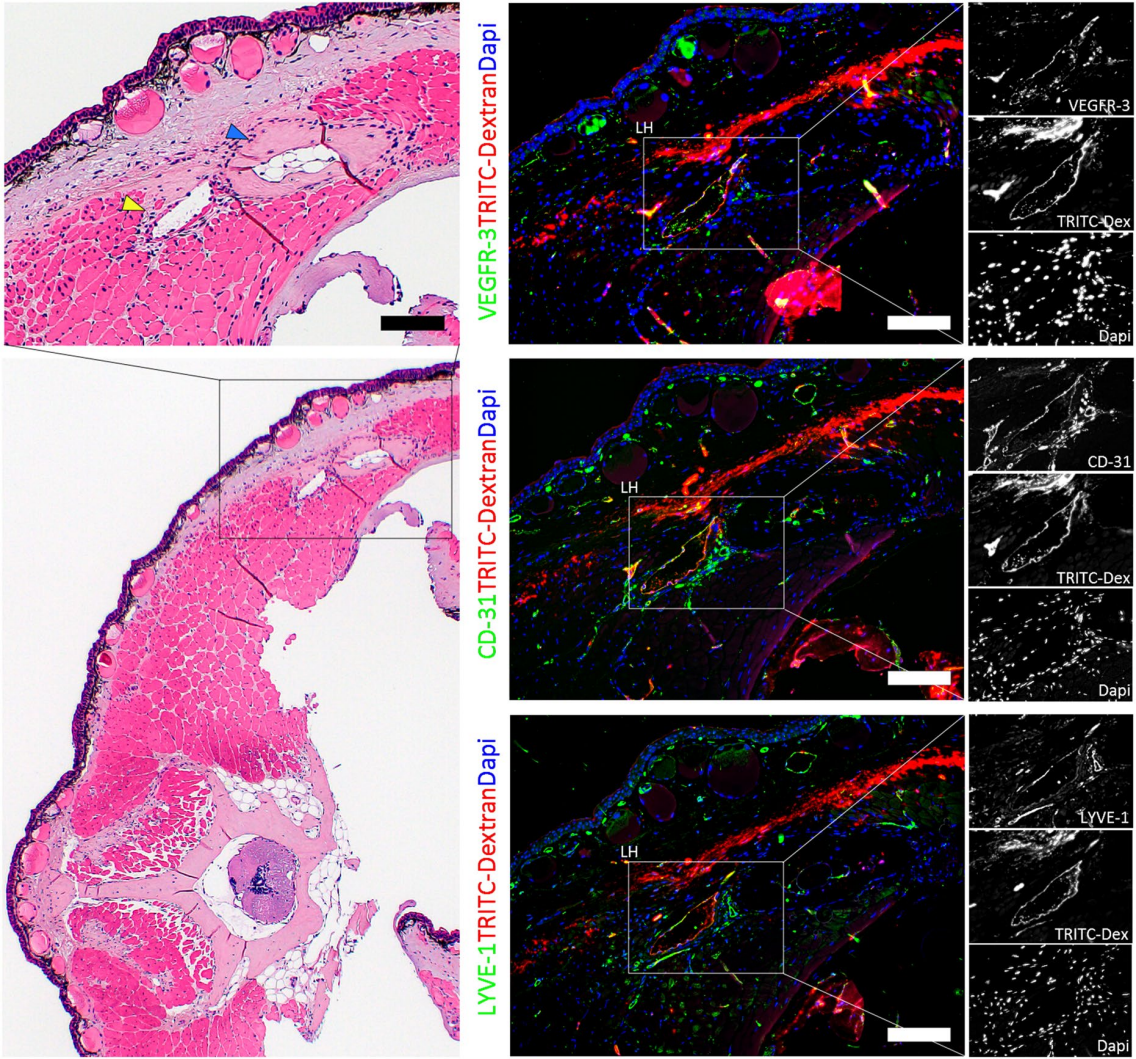
Lymphatic endothelial cell marker expression in newly regenerated lymphatics. Histological sections showing the regenerated lymphatic heart (LH) (yellow arrowhead) and the adjacent rib bone (blue arrowhead) in the abdominal region 120 days post complete microsurgical excision. The newly regenerated LHs had identical shape, size, structure, position, and function as the original LH. In addition, the newly regenerated LH demonstrated strong expression of lymphatic endothelial cell markers VEGFR-3 and LYVE-1 and pan-endothelial marker CD-31. The uptake of rhodamine-dextran dye confirmed the lymphatics were fully functional (Scale bars = 200 μm).

To confirm if the LHs regenerate we performed serial in-vivo microsurgical dissection, UHFUS and micro-CT lymphangiography following complete excision of single and multiple LHs. Regeneration consistently followed 5 morphological stages (Fig. 6a–e). Following resolution of the clot, robust angiogenesis and restoration of the structure of the major blood vessels was first observed in the first 28–60 days PO (Fig. 6a–e). This was followed by lymphangiogenesis 50–80 days PO (Fig. 6e,f) and complete regeneration of the LHs 100–130 days PO (Fig. 6g) with the more caudal tail LHs taking slightly longer to fully regenerate and resume pumping than the more cranial trunk LHs. The regenerated LHs had identical size, shape, structure, location, and function as the original LH including fully functional valves and flow, demonstrated by the uptake of rhodamine-dextran dye (Figs. 6g and 7). The newly regenerated lymphatic endothelial cells (LEC) had strong expression of VEGFR-3, LYVE-1 and CD-31 (Fig. 7).

### Lymphatic heart excision does not affect the rate of regeneration and the morphology of regenerated limbs

**Figure 8:**
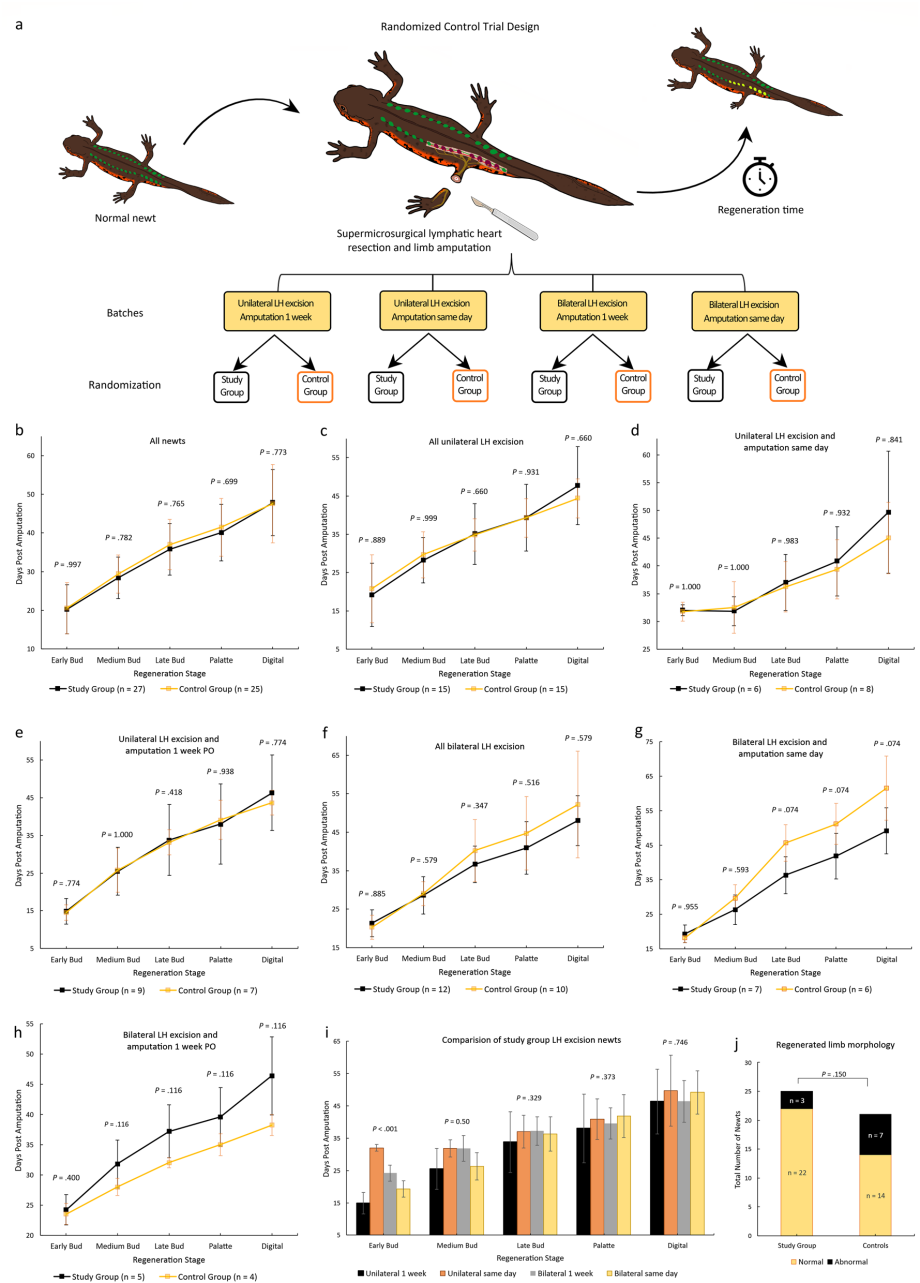
Randomized control trial evaluating the result of lymphatic heart excision on the rate and morphology of limb regeneration. (**a**) The randomized control trial design: n = 62 newts were enrolled and separated into 4 blocks, each block was randomized into a study and control group by a blinded independent assistant. Follow-up was complete in 52 newts, 10 newts died immediately postoperative. (**b**) There was no statistically significant difference in the overall rate of regeneration between study and control groups (Kolmogorov–Smirnov Z = 0.662, p = 0.773). (**c**–**h**) Subgroup analysis also showed no statistically significant difference in regeneration rates between study and control newts after unilateral LH excision (Kolmogorov–Smirnov Z = 0.730, p = 0 .660), regardless of the timing of amputation immediate amputation after LH excision (Kolmogorov–Smirnov Z = 0.617, p = 0.841) and amputation delayed 1-week post LH excision (Kolmogorov–Smirnov Z = 0.661, p = 0.774). Similarly, no significant difference between study and control newts was found after incremental bilateral LH excision (Kolmogorov–Smirnov Z = 0.778, p = 0.579) regardless of the timing of amputation immediate amputation (Kolmogorov–Smirnov Z = 1.284, p = 0.074) and amputation delayed 1-week PO (Kolmogorov–Smirnov Z = 1.193, p = 0.116). (**i**) Comparison of LH excision study newts from the different experimental batches (excluding controls) also showed no significant difference in the time taken to complete regeneration (Kruskal–Wallis H(3) = 0.1.229, p = 0.746). (**j**) Comparison of the morphological abnormalities in the regenerated limbs showed no statistically significant difference (p = 0.150) between study and control newts. Data represent mean ± standard deviation (error bars).

A randomized controlled trial was performed to evaluate the function of LH lymphaticovenous connections in limb regeneration using the hindlimb lymphatic model. Incremental supermicrosurgical excision of LH9 to LH15 was performed in study newts and a sham operation in controls followed by left hindlimb amputation (Fig. 8a). The two groups had similar mean snout-to-tail length, study group (101.55 mm, SD 4.57) and controls (101.97 mm, SD 4.82) t(60) = − 0.352, p = 0.726, and similar mean body weight, study group (5.37 g, SD 1.22) and the controls (5.45 g, SD 1.17) t(60) = − 0.245, p = 0.808. All newts received the assigned experimental treatment. Follow-up was complete in 52 newts with 10 newts, 4 study and 6 controls, unable to completely recover from anesthesia and died following surgery but this difference was not significant p = 0.732. There was no significant difference in the time taken to reach each of the regeneration stages between the study newts and controls. The mean number of days to reach the digital outgrowth stage endpoint was 47.81 days (SD 8.56) in the study group and 47.52 days (SD 10.137) in the controls, p = 0.773 (Fig. 8b). Further sub-group analysis showed no statistically significant difference between unilateral and bilateral LH excision, and between amputation on the same day as LH excision and 1-week delayed amputation (Fig. 8c–i).

Fewer newts in the study group developed anomalies in the regenerated limbs compared to controls however this difference was not statistically significant (Fig. 8j). The commonest limb anomalies observed were duplications of digits (n = 6 newts) and missing digits (n = 4 newts).

## Discussion

Lymphatic reconstructive microsurgery of lymphedema has established that much like the angiosomes of blood vessels, lymphatic vasculature despite its extensive inter-connections, is also organized into functional units with specific lymphatic collectors preferentially draining a specific 3-dimensional block of tissue territory also known as a lymphosome^28–30^. Defining these territories is essential for understanding the functional anatomy, embryological development, microsurgical treatment and for experimental modelling of the lymphatic system. In this study, we evaluated the functional lymphatic anatomy of the newt and revealed a novel lymphatic network inside the bone marrow connecting the superficial and deep lymphatic networks. We used our model and precision supermicrosurgery to show that the multiple LH lymphaticovenous connections which are an important distinguishing feature of the lymphatic and immune systems of newts and other highly regeneration competent vertebrates (Supplemental Fig. i) are not essential for regeneration. Furthermore, we demonstrated that newts are capable of regenerating fully functional LH after complete excision in a characteristic stepwise process. Therefore, newts provide a useful model for investigating both the role of lymphatic vasculature in regeneration and the regeneration of lymphatics. However, the notable anatomical differences between urodeles, anurans and mammals found in this study must be considered in the translation of results.

Historically, lymphatic research has lagged behind that of blood vessels because lymphatics are generally smaller, difficult to visualize without the use of contrast dyes and share many molecular markers with blood vessels which made them difficult to discriminate before the advent of LEC markers Prox-1, VEGFR-3, LYVE-1 and podoplanin. Panizza was the first to study lymphatics in salamanders in 1883 using mercury as a contrast agent, but this was heavily criticized for causing deformation of larger lymphatic spaces because of its weight and was replaced by liquid and semi-gelatinous dyes^9,10^. These methods were unsuitable for use in vivo and revealed little about the functional lymphatic anatomy. We used ICG NIRF as our primary modality complemented with UHFUS and micro-CT lymphangiography to define the lymphatic territories of the forelimb, hindlimb and the tail of the newt in-vivo and validated our model by showing decreased lymphatic to vein flow after LH excision. These regions were selected to create lymphatic models because they are the most widely used in regeneration research. A major limitation of NIRF we observed in both newts and frogs is their dark skin pigments absorb near-infrared light. Hence, we had to use transgenic albino animals which subsequently limited our sample sizes.

Our findings showed that the urodele lymphatic system is considerably different from the anurans. Although the 60.40% EF we found in newt LHs was comparable to the 20 to 80% reported in anurans, newt LH rates and EF did not show wide variation with the animal’s state of alertness^26,31^. The resulting 10 ml.Kg^−1^ min^−1^ total combined output of all 16 LH pairs in newts was also twice the estimated 0.9–5 ml.Kg^−1^ min^−1^ flowing through all the anurans LHs^26^. Paradoxically, unlike anurans, neither full general anesthesia nor complete excision of some or all the LHs in the newts resulted in lymphatic dysfunction or death, with collateral flow rapidly mobilized instead and circulation restored. This recruitment of collaterals following interruption of some major lymphatic collectors is shared by most other vertebrates, except anurans. In this respect, the urodele lymphatic system anatomically shares more similarities with other vertebrate classes than that of the adult anurans. Furthermore, we showed that newts do not possess the large subcutaneous lymphatics sacs characteristic of the adult anurans^10,32^. The result of the unique biology in anurans means their lymphatic flow is highly reliant on muscle movement and lung ventilation for flow of peripheral lymph into the lymphatic sacs and are equally dependent on their LHs for drainage of lymph into blood circulation. Thus, the anuran lymphatic system represents a highly specialized diversionary adaptation to meet the unique anuran body architecture needs rather than the exclusive amphibian model of evolutionary progression.

Strikingly, we identified a novel intraosseous network of lymphatics in the bone marrow of normal newts connecting the superficial lymphatic network, the larger deep vertebral plexus and the LHs. The preferential passage of these vessels through the bone cavity provides mechanical protection against collapse of the vessel lumen. This extensive network maintained lymphatic circulation even after removal of all the LHs, preventing lymphatic dysfunction in newts. Notably, we were unable to utilize Prox-1 expression for identification of LECs despite multiple attempts under varying experimental conditions. Therefore, we adopted a multimodal approach combining serial section computer 3D reconstruction with Micro-CT lymphangiography supported by endothelial cell VEGRF-3 and LYVE-1 expression analysis, TRITC-dextran dye injection studies and TEM analysis to comprehensively demonstrate the presence of a lymphatic vessel network in the vertebral bones and rib bones (Figs. 3 and 4). However, Prox-1 staining is still required to definitively prove the lymphatic identity of the VEGFR-3+ and LYVE-1+ vessels in the vertebral, rib, and long bones (Supplementary Fig. v).

Despite being a key primary lymphoid organ of the lymphatic system in many vertebrates, lymphatic vasculature in bone has only been identified in disease states including vascular bone tumors and extensive primary and secondary malignant bone tumors^33–35^. The mammalian bone aversion for lymphatics is most dramatically highlighted in Gorham-Stout disease, a poorly understood disorder characterized by the appearance of lymphatic vessels in bone leading to highly destructive and progressive bone resorption resulting in the affected bone vanishing altogether^36^. Our results show that despite the presence of lymphatics, the adult newt bone is neither a primary nor a secondary lymphoid organ^37,38^, but instead functions as an important lymphatic drainage organ and a fat reservoir in newts. We speculate that the lymphatics may additionally function in metabolism and mobilization of this bone marrow fat store.

Unlike the innate immune system, the role of the adaptive immune system in newt regeneration is poorly understood. Analyses of *scRNA-seq* in axolotl have consistently identified T and B cell markers *tac* and *igll5* at critical stages of limb regeneration^39–41^. However, our result showing no significant changes in regeneration after excision of major components of the newt lymphatic system vasculature, consistent with previous reports on excision of the largest secondary lymphoid organ, the spleen, strongly suggest the adaptive immune response my not be essential for regeneration but may instead have a regulatory role^42^.

Lymphedema is the direct result of impaired lymphatic vasculature function. The precise mechanisms behind failure of lymphatic regeneration following injury by surgery and chemoradiation remain unknown^43^. *TGF-β*_*1*_ mediated scarring and fibrosis as well as sclerosis and loss of pumping activity of collecting lymphatics are recognized as precipitating steps in the pathophysiology of lymphedema^43,44^. Asides the LH, amphibian lymphatic vessels lack both the muscular pulsating walls and valves seen in mammalian collecting lymphatics^45^. This attribute makes LHs ideal for modelling the structural and functional aspects of human collecting lymphatics. Notably, unlike the smooth muscle found in mammalian collectors the newt LH musculature shares the characteristics of skeletal and cardiac muscle^13,46^. We showed that newts are capable of regenerating LH after complete excision. The high expression of VEGFR-3 in the newly regenerated LECs suggests that like mammals, lymphatic regeneration in newts may also be dependent on the VEGF-C/VEGFR-3 pathway^47^. We speculate that damage of nerve supply may be the reason why the more caudal LHs had delayed resumption of pumping activity. Studies in frogs showed that LHs pumping takes 20–30 days to recover following denervation^46^. The observed regeneration of blood vasculature preceding lymphatics is reminiscent of embryological development of *Prox-1* expressing lymphatics sprouting from blood vessels^48,49^. Future studies should focus on the source of the LECs in the regenerating LHs, the differential spatiotemporal expression of Prox-1, LYVE-1, VEGFR-3, SOX18 and other factors associated with lymphangiogenesis, as well as the factors that influence the full recovery of pumping activity.

Clinically, microsurgical creation of lymphaticovenous anastomosis (LVA) either alone or as efferent LVA in vascularized lymph node transfers are now widely considered the main surgical treatment of lymphedema and lymphatic flow disorders^50–53^. These function much like the natural LH lymphaticovenous connections of newts. Therefore, we focused our study on these connections because they present a readily available opportunity for surgical translation of possible therapeutic benefits in patients beyond fluid circulation. The lack of association of lymphaticovenous connections with regeneration found in this study offers preliminary insights into the biology of healing tissue following LVA. However, the close association of the newt LH lymphaticovenous connections with intraosseous bone lymphatics, which are naturally absent in humans, presents a consequential reminder of the differences in lymphatic physiology between newts and humans. Addressing the immunological and metabolic functions of this intraosseous lymphatic network may provide insight on the evolutionary absence of lymphatics in human bone, their role in disease, immunity, fat metabolism and possibly in regeneration.

## Method

### Animals

Wild-type and transgenic albino adult Japanese fire belly newts *Cynops pyrrhogaster* 90 to 120 mm snout-to-tail length were obtained from Tsukuba University Faculty of Life and Environmental Sciences^54^. Wild-type and albino South African clawed frogs *Xenopus Laevis* were obtained from a local pet store. Wild-type newts and all the frogs were housed at the Department of Plastic Surgery, Mie University while the albino newts were housed at the Faculty of Life and Environmental Sciences, Tsukuba University. All experiments involving animals were performed in accordance with the guideline and regulations approved by the Mie University Animal Care Committee and the Animal Care and Use Committee of the University of Tsukuba Approval (Registration Code 170110). All methods were performed and reported in compliance with the ARRIVE guidelines 2.0^55^.

### Anesthesia

Animals received anesthesia with 0.1% FA100 solution (4-allyl-2-methoxyphenol; DS Pharma Animal Health, Osaka, Japan) in water at room temperature for 1–2 h before experimental procedures^56^.

### Near infrared fluorescence lymphangiography

ICG NIRF lymphangiography was performed on transgenic albino newts^54^ (n = 9 newts) and albino frogs (n = 3 frogs). Subcutaneous injection using a 34G needle of 10–20 μL of a 1:1000 solution Diagnogreen 25 mg/ml (Daiichi Sankyo, Tokyo, Japan) diluted with sterile water for injection was used to evaluate lymphatic flow under normal physiological conditions and 50 μL was used to evaluate excess tissue fluid drainage. The injections of the dorsal and ventral surfaces of the hand (forelimb) and foot (hindlimb), and the left and right sides of the distal 10–15 mm of the tail were each assessed separately. Lymphatic flow was visualized and recorded using a PDE Neo NIRF camera (Hamamatsu Photonics, Hamamatsu City, Japan) at high magnification held 15–25 cm away from the animals.

### Immunohistochemistry and fluorescent dye studies

Immunohistochemistry was performed as previously described by Casco-Robles et al.^57^. Briefly, samples were washed in PBS, 0.2% TritonX-100 in PBS and again in PBS for 15 min each, incubated in blocking solution (2% normal goat serum (S-1000; Vector Laboratories, Burlingame, CA, USA)/0.2% TritonX-100 in PBS) for 2 h, washed as before, and then incubated in primary antibody diluted in blocking solution. For VEGFR-3 staining tTBS was used in place of PBS. Fluorescence dye studies were performed using Tetramethylrhodamine-dextran 2,000,000 MW (Invitrogen D-7139) reconstituted in sterile water for injection injected subcutaneously 20–50 μL and tissue samples harvested 10–15 min later. Lymphatic vessels were identified and differentiated from blood vessels by comparing differential rhodamine-dextran uptake and differential staining of VEGFR-3, LYVE-1 and CD-31. LECs were identified as VEGFR-3 + , LYVE-1 + , CD-31 + and BEC identified as CD-31 + , LYVE-1 ± , VEGFR-3-. Additional identification of lymphatic vessels by staining with commercially available Prox-1 antibodies in mature adult newts was attempted under varying experimental conditions however this was unsuccessful (data not shown). Histology images were captured using a Keyence BZ-X710 Microscope and An Olympus AX80 coupled with an Olympus DP74 Microscope Digital Camera. Image processing was performed using Image J^58,59^.

### Antibodies

The following antibodies were purchased, Anti-LYVE-1 Novus (NB600-1008), Anti-CD-31 Bioss (BS-0195R), Anti-LYVE-1 Abcam (Ab14917). Anti-VEGFR-3 antibody was custom made based on the newt nucleotide orthologue by Eurofins Japan. Further details on the antibodies used are provided in the supplementary data.

### Lymphatic heart microsurgical excision procedure

Following anesthesia, 50 μL ICG dye was injected subcutaneously into the limbs or tail of the newts and frogs. A longitudinal skin incision was made on the back immediately inferior to the lateral line ridge overlying the LHs to be removed using a No. 15 surgical scalpel and a 2–3 mm wide skin flap was raised in the subdermal plane using supermicrosurgery dissection technique and OMS 800 and OMS 610A operating microscopes (Topcon, Tokyo, Japan) to preserve skin blood vessel perforators. The LHs were identified visually by pulsations and collection of ICG dye. LH were excised using supermicrosurgical technique and supermicrosurgery titanium instruments (EMI Factory CO., Ltd. Nagano, Japan) to carefully preserve surrounding tissue in the study group while in the controls identical surgery was performed but the LHs were left intact. The skin flaps were closed using interrupted nylon 10/0 sutures.

### Randomized control trial of limb regeneration after lymphatic heart excision

A total of 62 newts were enrolled into the randomized control trial and divided into 4 blocks designed to evaluate regeneration after incremental LH excision and regeneration after the onset of circulatory changes at 1 week PO. Newts were then randomized into approximately equal experimental groups using SPSS 26 by a blinded and independent assistant (from HPB Surgery Department). Independent *T* tests were used to compare the mean snout-to-tail length and weight of the two groups. The 7 LHs draining the lower limb were identified in all newts and excised using supermicrosurgical technique as described above in the study group while in the controls identical dissection was performed but the LHs were left intact. The left hindlimb was amputated 2 mm proximal to the knee joint using a No. 10 surgical scalpel and the protruding femur trimmed flush with the wound. Gentle pressure was applied for a few min to achieve hemostasis. Blinding of the investigators was not possible due to the surgical nature of the experimental treatment. However, to limit bias, the assigned group was revealed to the microsurgeons only after wound exposure and lymphatic microdissection. The newts were kept unfed in separate plastic containers at room temperature, observed daily and the morphological stage of regeneration recorded^60^. Our original protocol planned for the assessment of the limb regeneration time by 3 blinded investigators. However, we departed from the protocol due to limited staff availability between 2020 and 2021 and instead, a single unblinded investigator performed this assessment. The rate of regeneration was compared between study and control groups using the Two-Sample Kolmogorov–Smirnov test. The Kruskal–Wallis test was used for subgroup analysis of the study group LH excision newts. Assessment of regenerated limb morphology was performed by 2 blinded investigators. Fisher Exact Test was used to compare the proportions of morphological anomalous regenerated limbs and PO deaths.

### Measurement of Edema after lymphatic heart removal

In newts (n = 6 study group newts, n = 5 control newts) the left hindlimb diameter was measured at 3 points; 3 mm proximal to the ankle joint, at the knee joint and in the thigh 2 mm proximal to the knee 1 week post LH excision by 2 independent, blinded assistants. Intraclass Correlation Coefficient was calculated based on a mean-rating (k = 2), absolute-agreement, 2-way mixed-effects model. For frogs (n = 2 frogs), the frogs were patted dry with a paper towel to remove excess water and the total body weight was measured using a digital microbalance.

### Fluorescence contrast analysis of lymphatic to vein flow

The density of rhodamine-dextran positive intramuscular vessels was assessed by counting the number of vessels in a 20 × field on 24 to 36 different paraffin cross-sections of the base of the tail, abdomen and chest of each newt. N = 3 newts were assessed 28 days post excision of all LHs, n = 1 newt assessed after 120 days and n = 3 control newts 28 days and 120 days after a sham operation. Independent *T* tests were used to compare the vessel densities between groups.

### Micro-CT lymphangiography

Micro-CT lymphangiography was performed using CosmoScan GXII (Rigaku, Tokyo, Japan) before and 10–60 min after subcutaneous injection of 10–50 μL Iohexol (Omnipaque®300, GE Healthcare Pharma, Tokyo, Japan) and the images processed using RadiAnt DICOM Viewer software. Quantitative analysis of the VCP was performed using Image J^59^. The mean grey value of the cross section of the VCP was measured at 6 different points along its course from the pelvis to the liver in each newt and an independent T-test used to compare study (n = 3 newts) and control (n = 3 newts) groups.

### Ultra-high frequency ultrasonography (UHFUS)

UHFUS was performed on 6 newts (n = 2 normal newts, n = 2 LH resection newts, n = 2 control operation newts) using VeVo MD UHF70 (FUJIFILM Visualsonics, FUJIFILM, Tokyo, Japan) before and after injection of 20–50 μL saline into the limbs. A total of n = 8 LH from 2 newts were used to calculate LH EF. A total of n = 3 LH from 2 study newts and n = 11 LH from 1 control and 2 normal newts were used for comparison of LH rate compensation after excision of the posterior LH using an independent *T* test.

### Morphological staging of lymphatic heart regeneration

For single LH excision (n = 5 newts) a 2 × 4×2 mm skin flap was elevated in the proximal tail and a single LH excised using supermicrosurgical technique as described above. Regeneration was assessed by opening the skin flap at weekly, then monthly intervals for 150 days. For multiple LH excision, n = 1 newt post excision of all 16 pairs of LH and n = 16 study group newts from the limb regeneration experiment were observed at 100 days to 150 days by opening skin flaps along the area where the LH were removed and using micro-CT lymphangiography. Tetramethylrhodamine-dextran lymphangiography and IHC were performed on serial histological sections at 28 days (n = 3 newts) and 120 days (n = 1 newt).

### Tissue fixation by trans-lymphatic perfusion (FixLyP)

FixLyP was performed to clear the red blood cells from blood vessels and fix the lymphatic vessels to prevent their collapse in preparation for computer 3D volume reconstruction of serial slides. A midline abdominal skin incision was made taking care not to penetrate the peritoneum. The VCP (diameter 0.3–0.5 mm) was identified as it ascends from the pelvis into the liver immediately deep to the peritoneum using an operating microscope. The peritoneum was opened, small size microvascular clamps applied proximally and distally, and the vein cut 5 mm before it enters the liver. The cephalic end of the vein was prepared using standard microsurgical technique to dissect off the adventitia. A microvascular round-tipped blunt vascular needle was then inserted into the cephalic end of the vein and the vascular clamps released. Cold heparin saline 4 °C was slowly injected into the vein using a 1 ml syringe allowing the caudal end of the vein to bleed freely until the liver color became whitish and clear heparin saline was seen draining. Next, cold 4% paraformaldehyde (PFA) 4 °C was slowly injected at 20–50 μL/Body weight (g)/min into the subcutaneous tissue of the limbs and tail until the newt’s body became rigid. Samples were then collected and placed immediately in cold 4% PFA for 4 h then demineralized using EDTA for 2 days.

### Serial section histology and 3-D computer volume reconstruction

Serial paraffin embedded sections 5 μm thick were stained with HE and scanned at 20 × using a NanoZoomer S360 Digital slide scanner (Hamamatsu Photonics K. K, Hamamatsu, JAPAN). Digital 3-D computer volume reconstruction of 900 serial slides of the abdomen and tail was performed using Image J (FiJi Version 1.53f51) TrakEM2 plugin^58,59^. In cases where a tissue section was heavily distorted or damaged by preparation artifacts, the image was excluded from the stack and the best fit adjacent image was duplicated to maintain the tissue volume. Lymphatic vessels were tracked and marked in a retrograde fashion while blood vessels were marked in antegrade from the LHs (Supplementary Fig. iv).

### Electron microscopy

Samples were prefixed in 2.5% glutaraldehyde for 2 h at 4 °C then demineralized using K-CX (Falma, Tokyo, Japan), washed 3 times in PBS for 10 min and postfixed in 1% OsO_4_ for 1 h. PBS wash was repeated twice for 15 min, and the sample dehydrated in increasing concentrations of ethanol then transferred to acetone. The samples were infiltrated then embedded in epoxy resin. Serial 400 μm scanning sections stained with toluidine blue were prepared for light microscopy and 80 nm sections cut for transmission electron microscopy then viewed using a JEM-1400Flash Electron Microscope (JEOL, Tokyo, Japan).

### Data analysis software

Analyses were performed using SPSS 26 (IBM Corp. Released 2019. Armonk, NY) and SPSS 27 (IBM Corp. Released 2020. Armonk, NY). All the statistical tests performed were 2-tailed test with p < 0.05 considered statistically significant.

### Conference presentation

Presented at the 31st Annual University of Tokyo Plastic Surgery Scientific Conference, January 2020, Tokyo, Japan.

## Supplementary Information

Supplementary Information.

Supplementary Video 1.

Supplementary Video 2.

## Data Availability

The data that support the findings of this study are available from the corresponding author upon reasonable request.

## Abbreviations

BEC: Blood vessel endothelial cells
ICG: Indocyanine green
NIRF: Near-infrared fluoroscopy lymphangiography
LEC: Lymphatic endothelial cell
LH: Lymphatic heart
UHFUS: Ultra-high frequency ultrasonography
TLyLL: Trunci lymphatici longitudinales lateralis
TLyLPab: Trunci lymphatici longitudinales parabdominales
TlyLPe: Trunci lymphatici longitudinales parepigastrici
TLyLSv: Trunci lymphatici longitudinales subvertebrales
TvIL: Transverse vertebral intraosseous lymphatic vessels
TvIV: Transverse vertebral intraosseous veins
VCP: Vena cava posterior
VL: Venous lateralis
