# Supplementary material for: Structural and functional analysis of the newt lymphatic system
Source: Sci Rep. 2023 Apr 27;13:6902. doi: 10.1038/s41598-023-34169-w (PMC10140069; doi:10.1038/s41598-023-34169-w)
Supplement: Supplementary file 1 — Supplementary Information. [file 41598_2023_34169_MOESM1_ESM.pdf]

STRUCTURAL AND FUNCTIONAL ANALYSIS OF THE NEWT LYMPHATIC SYSTEM

Supplementary Digital Content

Authors

Chihena H BANDA, M.D.[1], Makoto SHIRAISHI M.D.[1], Kohei MITSUI M.D.[1], Yoshimoto OKADA M.D.[1], Kanako DANNO M.D.[1], Ryohei ISHIURA M.D.[1], Kaho MAEMURA M.D.[1], Chikafumi CHIBA PhD. [2], Akira MIZOGUCHI M.D, PhD.[3], Kyoko IMANAKA-YOSHIDA M.D, PhD.[4], Kazuaki MARUYAMA M.D, PhD.[4], Mitsunaga NARUSHIMA M.D, PhD.[1]

1. Department of Plastic and Reconstructive Surgery, Mie University, Tsu, JAPAN
2. Faculty of Life and Environmental Sciences, University of Tsukuba, Tsukuba, Ibaraki, JAPAN
3. Department of Personalized Cancer Immunotherapy, Mie University, Tsu, JAPAN
4. Department of Pathology and Matrix Biology, Mie University, Tsu, JAPAN

MIE UNIVERSITY
DEPARTMENT OF PLASTIC AND RECONSTRUCTIVE SURGERY

Contents

Relationship Between the Lymphatic System to Blood Vascular System Connections and Regeneration Ability in Vertebrates 1
Extravascular Fluid Transport 3
Serial Histology and 3D Computer Reconstruction 4
Functional Lymphatic Networks of the Newt 10
Bone Marrow Histology 11
  Forelimb 11
  Vertebrae 14
Posterior Lymphatic Heart Excision in *Xenopus laevis* 15
Antibodies 16
  Commercially Purchased Antibodies 16
  Custom Made Antibody 17
References 18

Relationship Between the Lymphatic System to Blood Vascular System Connections and Regeneration Ability in Vertebrates

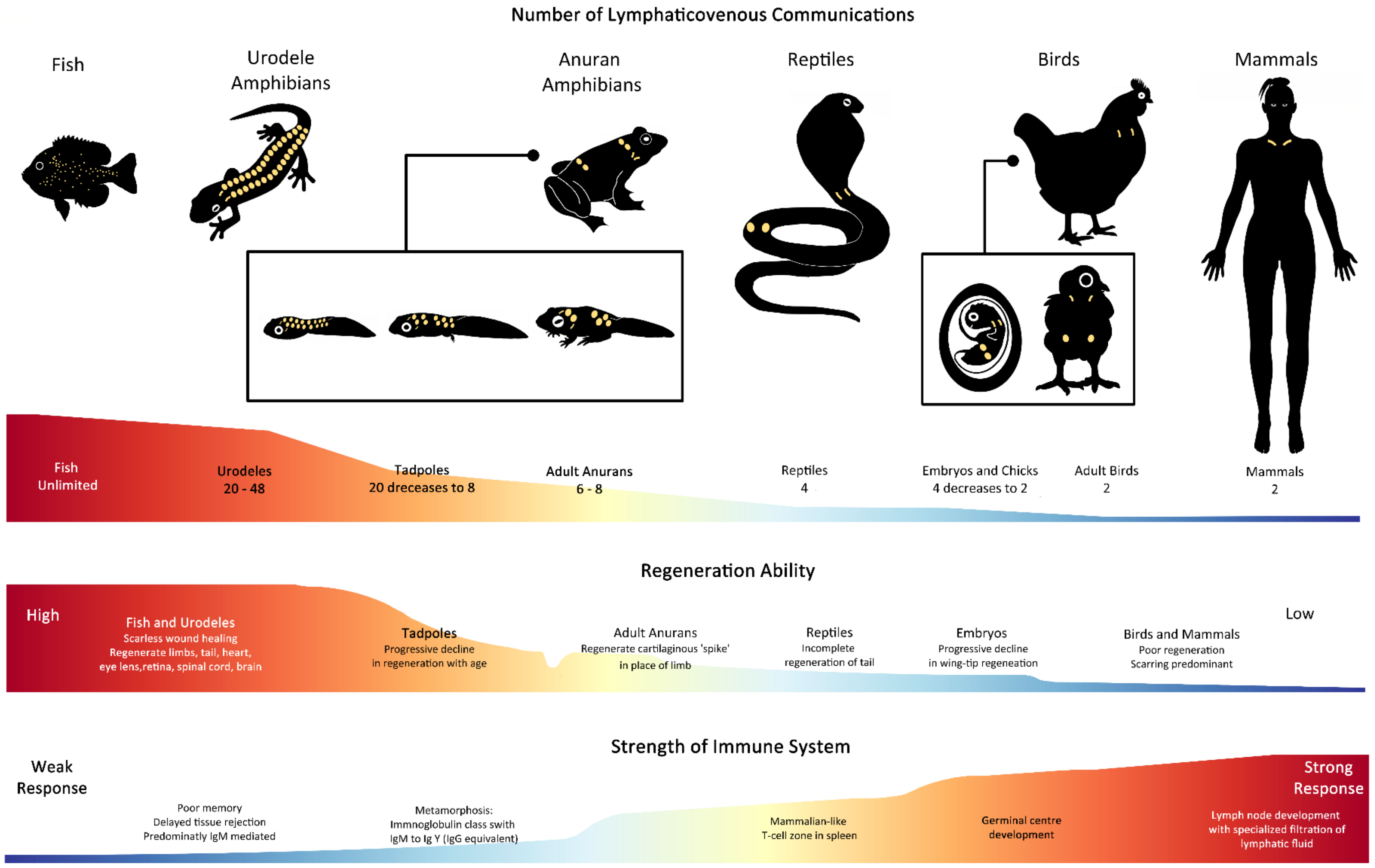

Supplementary figure (i): The Relationship Between Lymphatic System to Blood Vascular System Connections and Regeneration Ability in Vertebrates. The direct lymphatic vessel to venous (lymphaticovenous) connections are shown at the lymphatic hearts (yellow ovals) and at the thoracic lymphatic ducts to subclavian veins (yellow bars). The number of lymphaticovenous connections progressively decreases from lower vertebrates, fish and urodele amphibians, to mammals. In fish the secondary vascular system is extensively anatomically interconnected with the main blood vascular system and occasional blood flow is seen. Urodele amphibians (newts and salamanders) have 20 to 48 connections. In anuran amphibians (frogs and toads), larvae tadpoles have 20 lymphaticovenous connections that progressively decreases to 6 to 8 after metamorphosis into adults. Reptiles and birds have 4, that decreases to 2 in most birds in the early post embryonic period. Mammals have only the 2 lymphaticovenous connections located at the thoracic lymphatic ducts to subclavian vein junctions. The number of these lymphaticovenous connections is inversely correlated with the strength of the immune system and directly correlated with regeneration ability.

Extravascular Fluid Transport

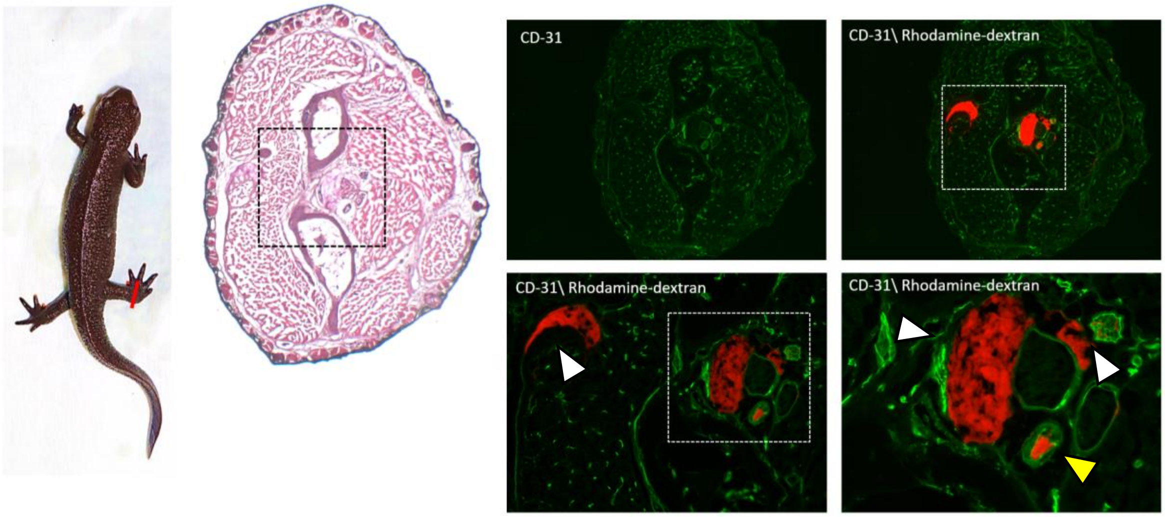

Supplementary figure (ii): Frozen sections 12µm thick of the newt lower leg following excess rhodamine-dextran injection of 0.05ml showing extravascular fluid transport (white arrows) in intermuscular septa and along nerve sheaths areas not enclosed in endothelium shown by the absence of pan-endothelial marker CD31 expressed by both blood vessels and lymphatic vessels. Intravascular transport of the dye is shown (yellow arrow) in a vessel lined by endothelium.

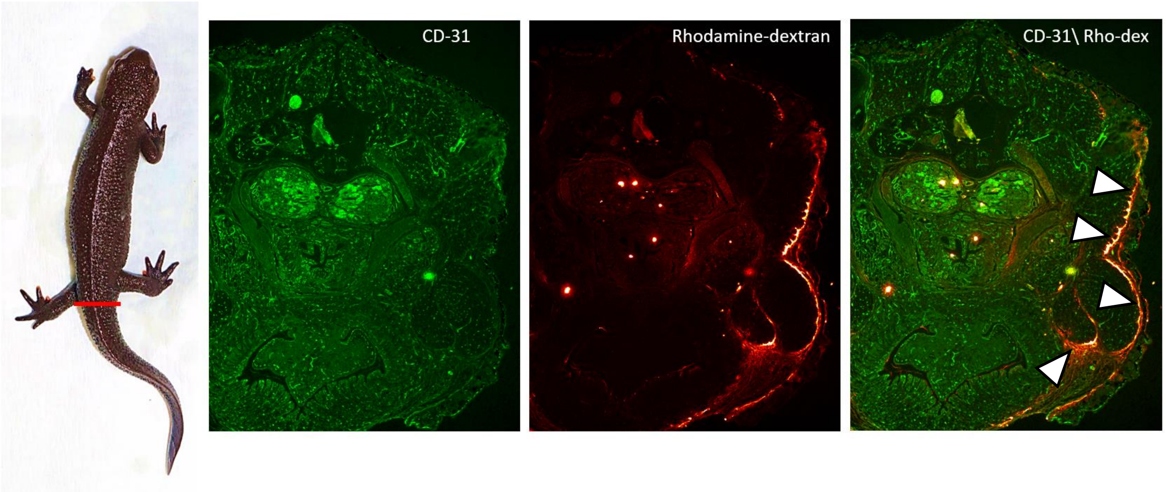

Supplementary figure (iii): Frozen sections 12µm thick of the pelvis following excess rhodamine-dextran injection of 0.05ml showing extravascular fluid transport (white arrows) in subcutaneous tissue and in intermuscular septa not enclosed in endothelium shown by the absence of pan-endothelial marker CD31 expressed by both blood vessels and lymphatic vessels.

Serial Histology and 3D Computer Reconstruction

Serial paraffin embedded sections 5µm thick were stained with HE and scanned at 20x using a NanoZoomer S360 Digital slide scanner (Hamamatsu Photonics K. K, Hamamatsu, JAPAN). Digital 3-D computer volume reconstruction of 900 serial slides of the abdomen and tail was performed using Image J (FiJi Version 1.53f51) TrakEM2 plugin. Lymphatic vessels were tracked and marked in a retrograde (green) fashion while blood vessels were marked in antegrade (red) from the lymphatic heart (LH). **Supplementary figure (iv)** demonstrates a section of the image stack showing the transverse intraosseous lymphatic vessel (TvIL) in an abdominal rib bone (blue arrowhead) flowing directly to the LH (yellow arrowhead) as the major input vessel. The transverse intraosseous vertebral vein (TvIV) connecting to the venous lateralis receiving the LH output is shown in red running intimately with the TvIL.

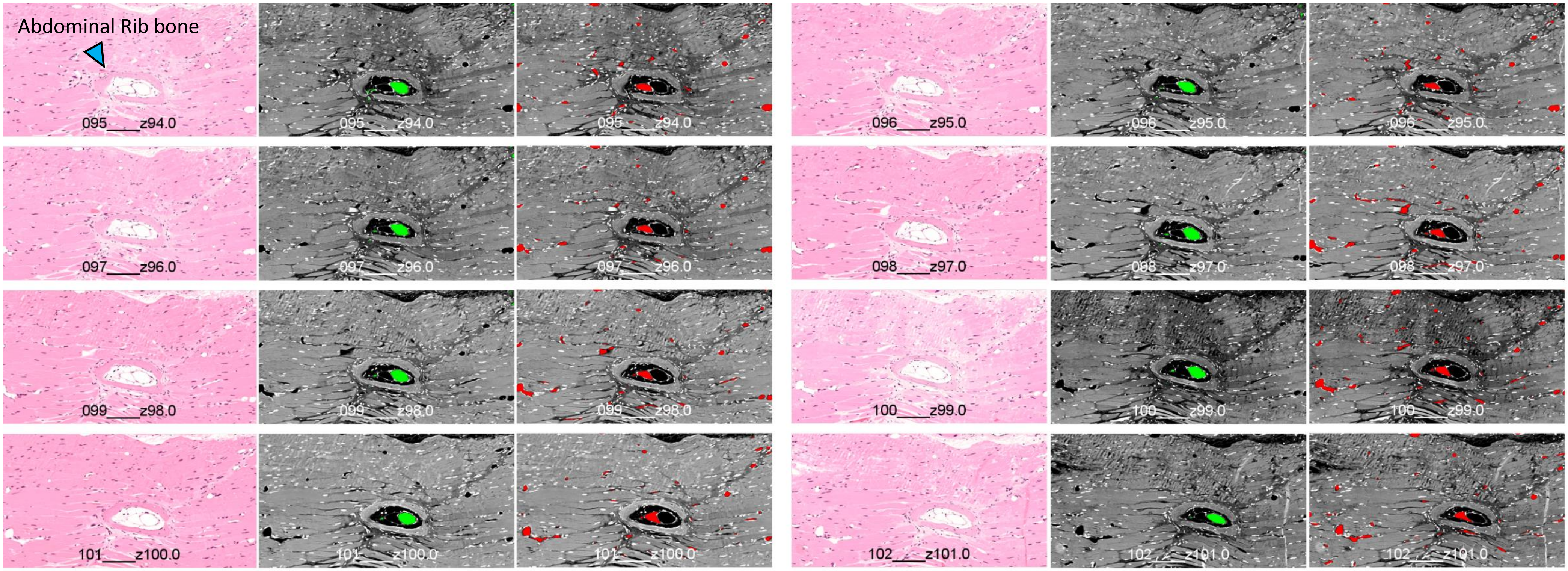

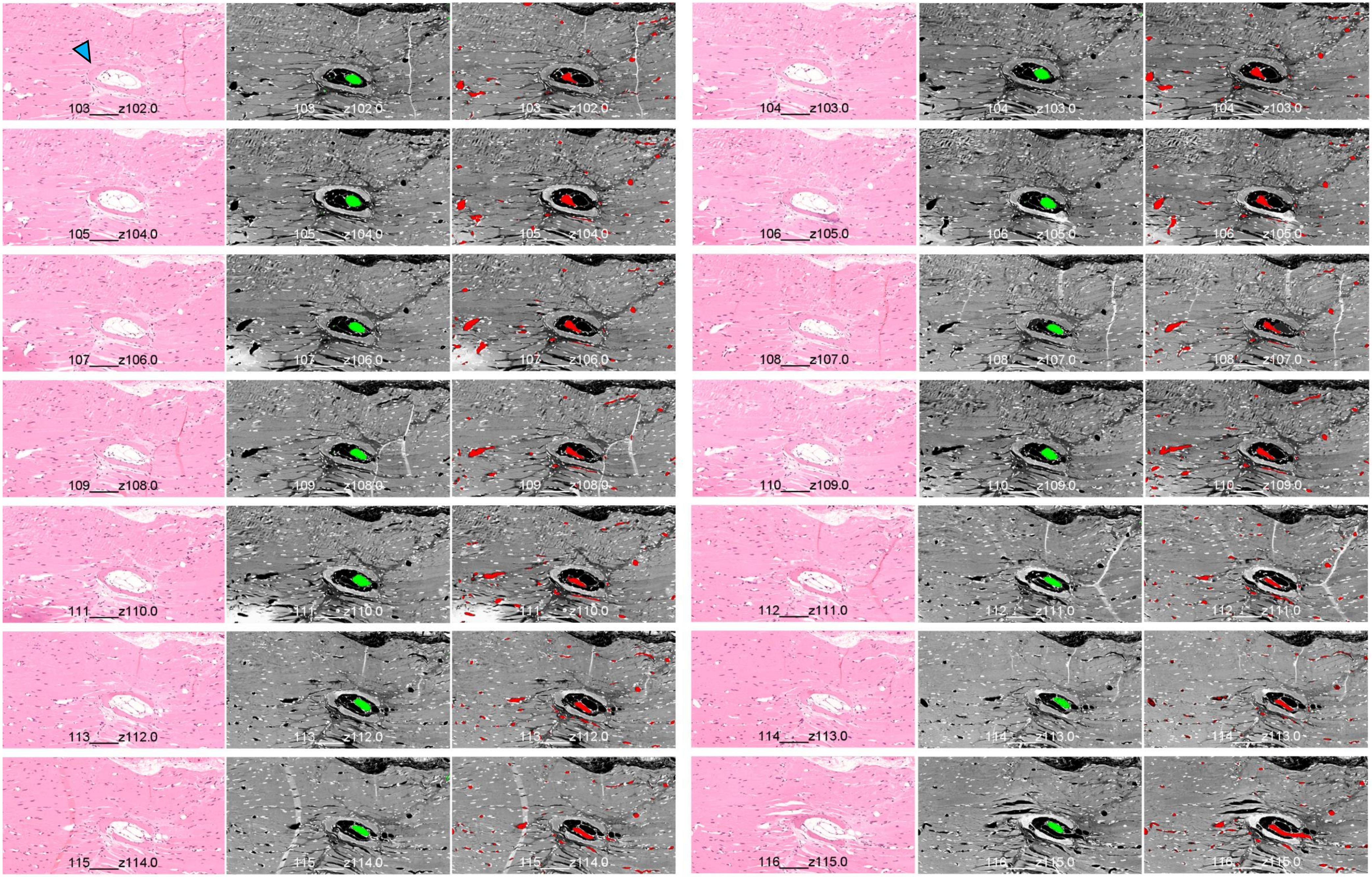

MIE UNIVERSITY
DEPARTMENT OF PLASTIC AND RECONSTRUCTIVE SURGERY

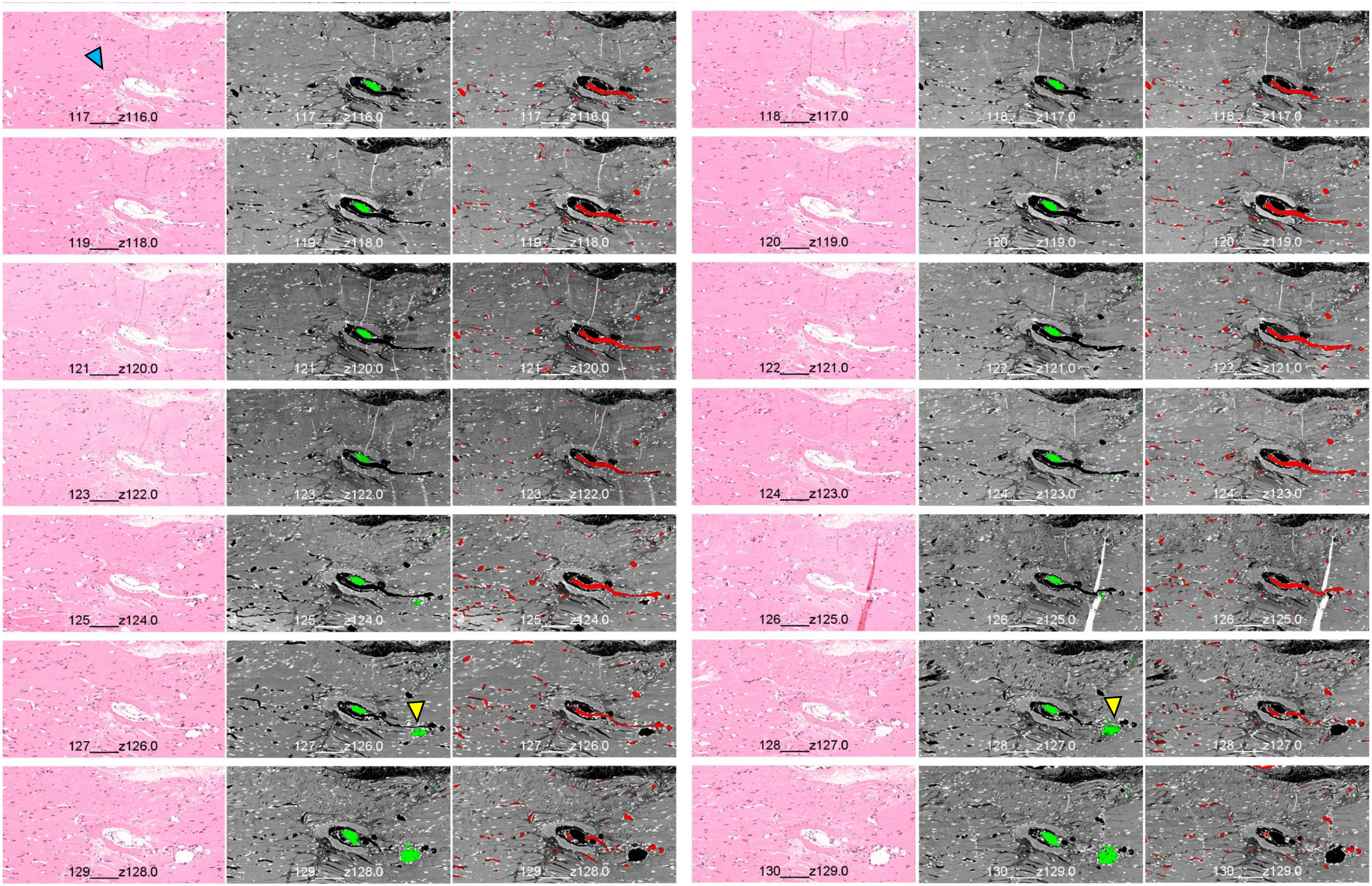
117____z116.0
118____z117.0
119____z118.0
120____z119.0
121____z120.0
122____z121.0
123____z122.0
124____z123.0
125____z124.0
126____z125.0
127____z126.0
128____z127.0
129____z128.0
130____z129.0

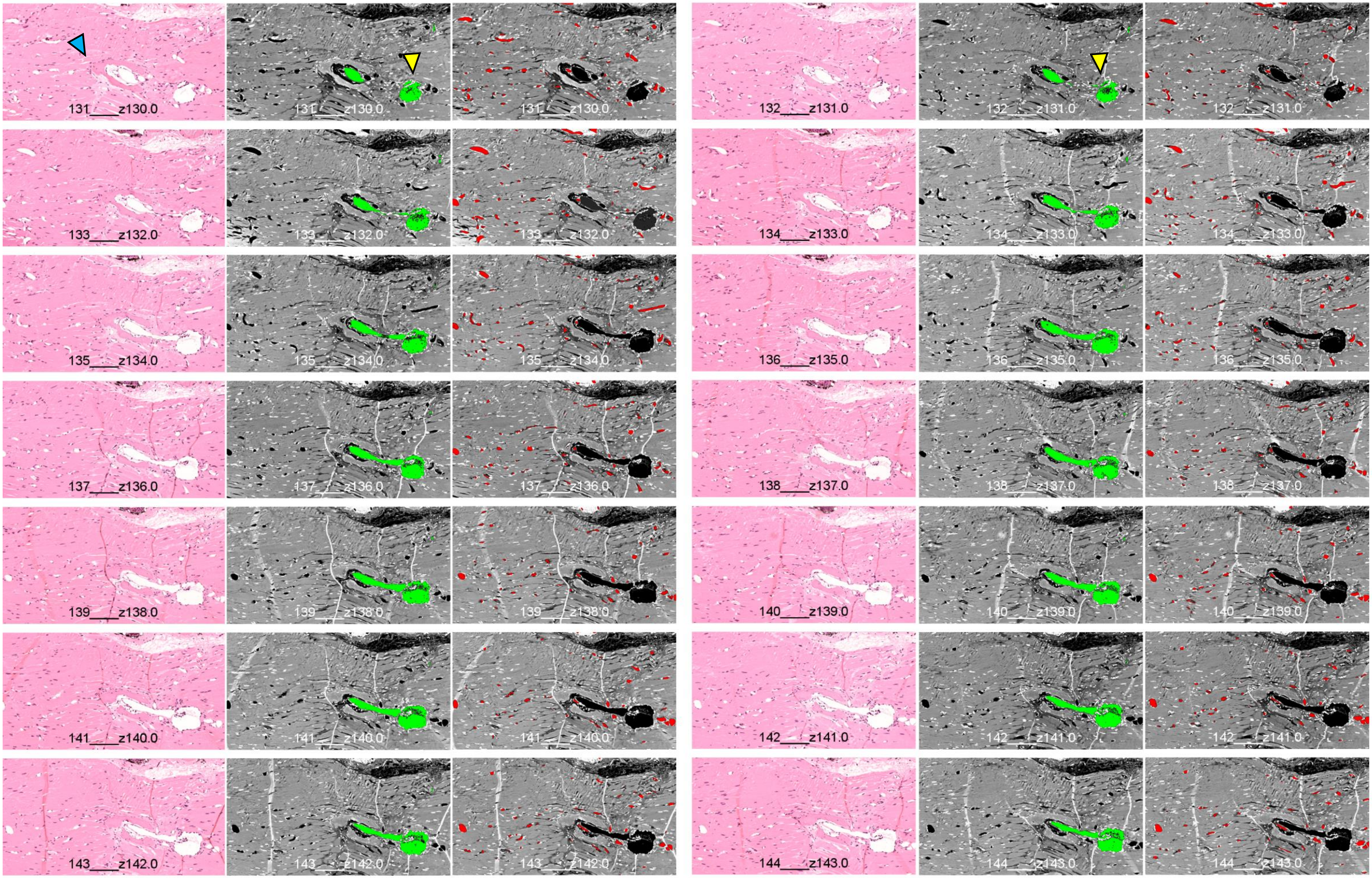
131___z130.0
131___z130.0
131___z130.0
132___z131.0
132___z131.0
132___z131.0
133___z132.0
133___z132.0
133___z132.0
134___z133.0
134___z133.0
134___z133.0
135___z134.0
135___z134.0
135___z134.0
136___z135.0
136___z135.0
136___z135.0
137___z136.0
137___z136.0
137___z136.0
138___z137.0
138___z137.0
138___z137.0
139___z138.0
139___z138.0
139___z138.0
140___z139.0
140___z139.0
140___z139.0
141___z140.0
141___z140.0
141___z140.0
142___z141.0
142___z141.0
142___z141.0
143___z142.0
143___z142.0
143___z142.0
144___z143.0
144___z143.0
144___z143.0

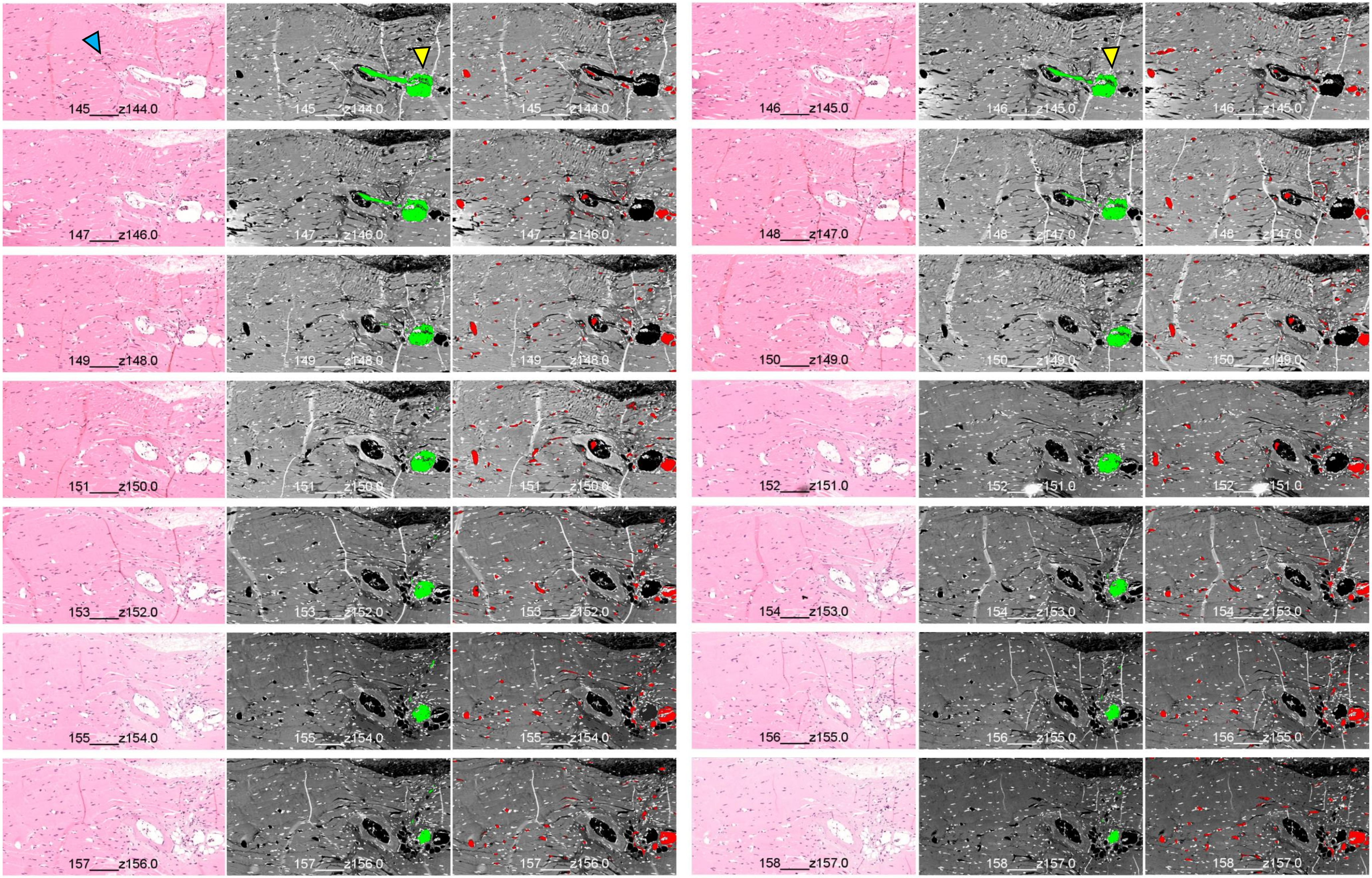

MIE UNIVERSITY
DEPARTMENT OF PLASTIC AND RECONSTRUCTIVE SURGERY

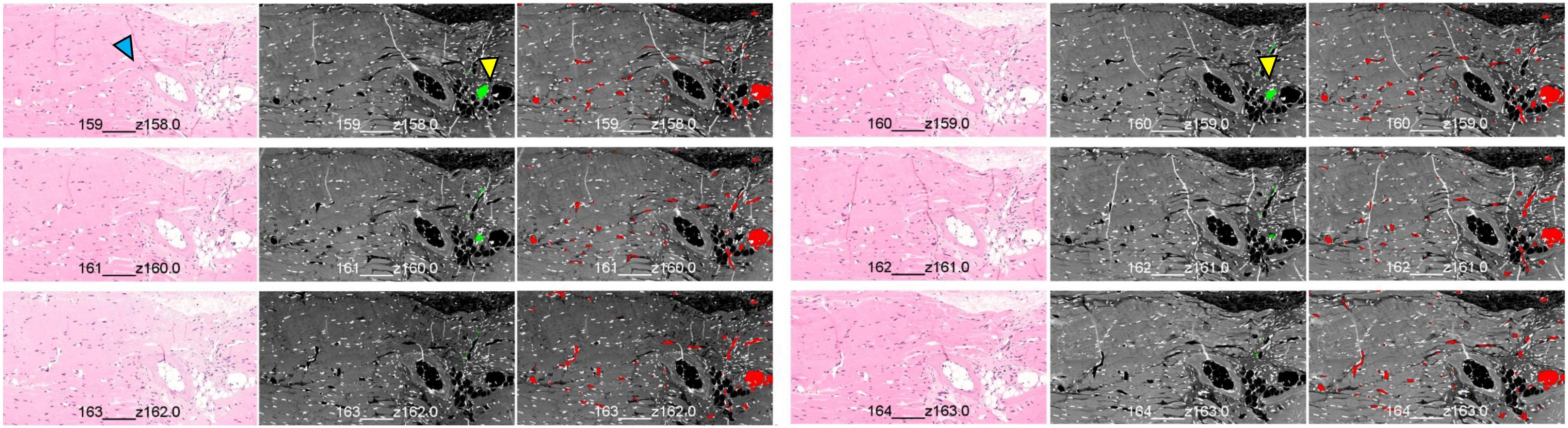

Supplementary figure (iv): Section of the Serial Histological Images used for 3D Computer Reconstruction showing a cross section of the lateral end of an abdominal rib bone (blue arrowhead) with the transverse vertebral intraosseous lymphatic vessel and transverse vertebral intraosseous vein inside the bone and both connecting directly to the LH (yellow arrowhead) forming the LH input and output respectively.

Functional Lymphatic Networks of the Newt

Superficial Lymphatic Network

- Formed by lymphatics in the dermal and subdermal layers.

- Included fine dermal lymphatics, body segmenta collectors, TLyPab, TLyLPe and TLyLL which were highly interconected and drained into LH via the TLyLL

- The muscle layer had few lymphatics but had rich network of blood vessels

TvIL connecting the 2 networks and draining to the LH

Deep Lymphatic Network

- Formed by the deep tissue and visceral lymphatics

- Included the TLyLSv and vertebral plexus of lymphaitcs

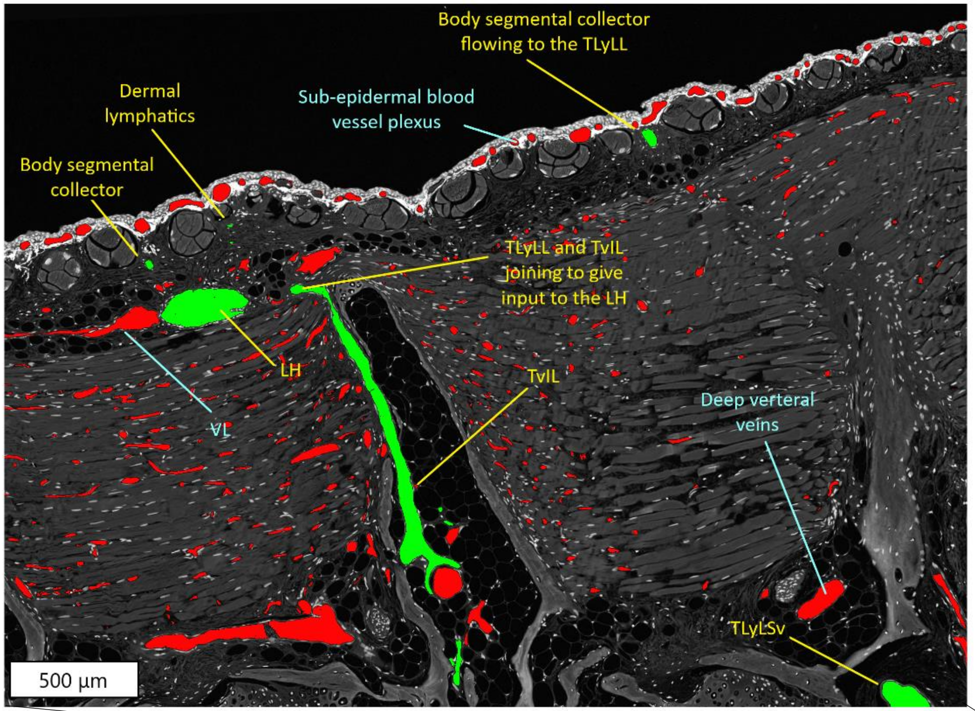

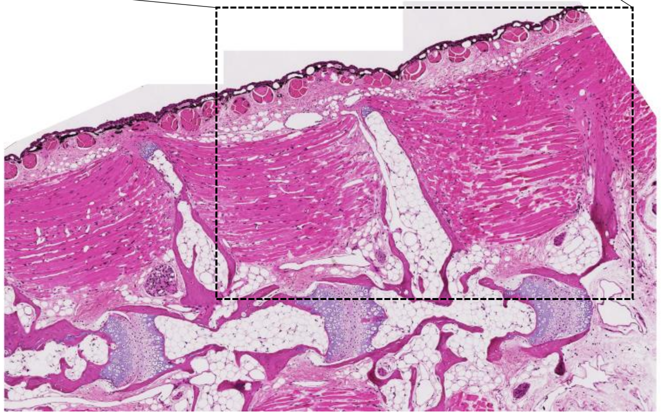

Supplementary figure (v): Functional lymphatic networks of the newt. Serial section computer 3D volume reconstruction of the newt proximal tail showing the superficial and deep lymphatic networks connected by the intraosseous lymphatics. LH = Lymphatic heart, TvIL = Transverse vertebral intraosseous lymphatic vessels, TLyLSv = Trunci lymphatici longitudinales subvertebrales, TLyLL = Trunci lymphatici longitudinales lateralis. VL = Venous leateralis

Bone Marrow Histology

Forelimb

HE

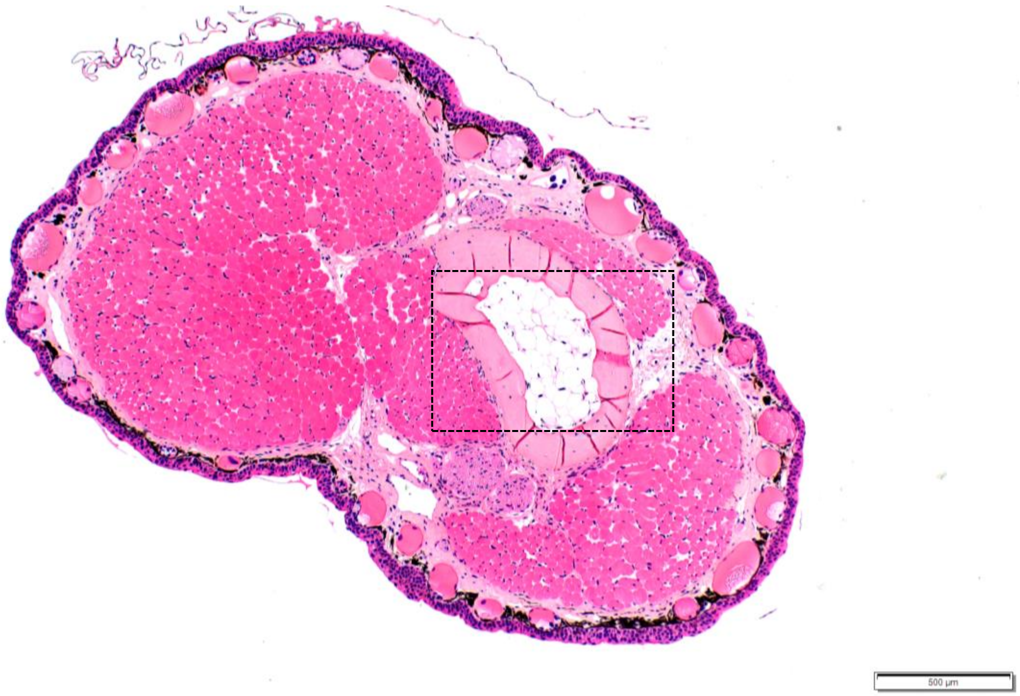

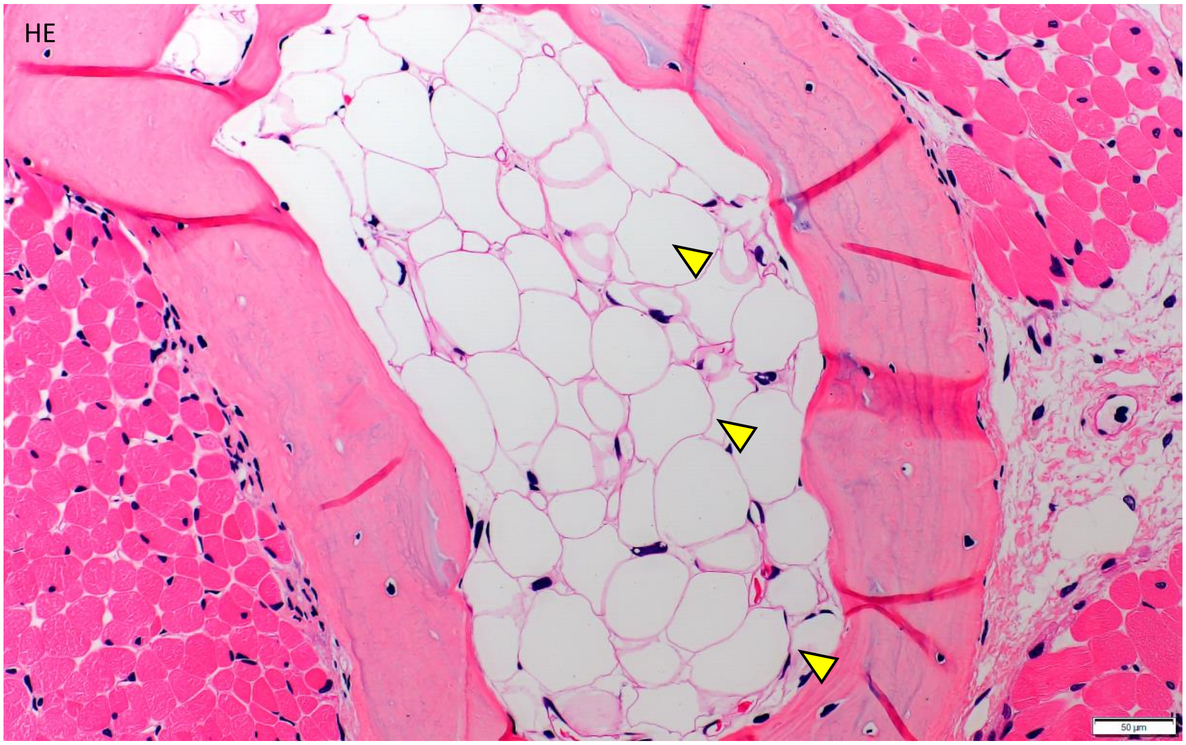

CD-31

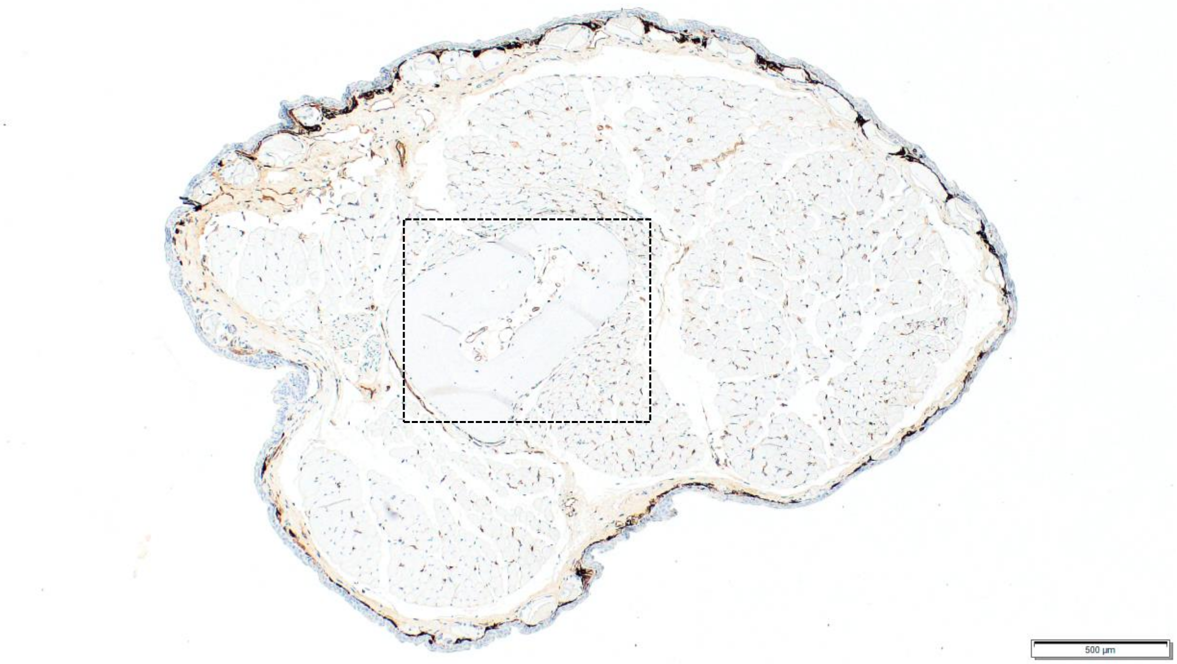

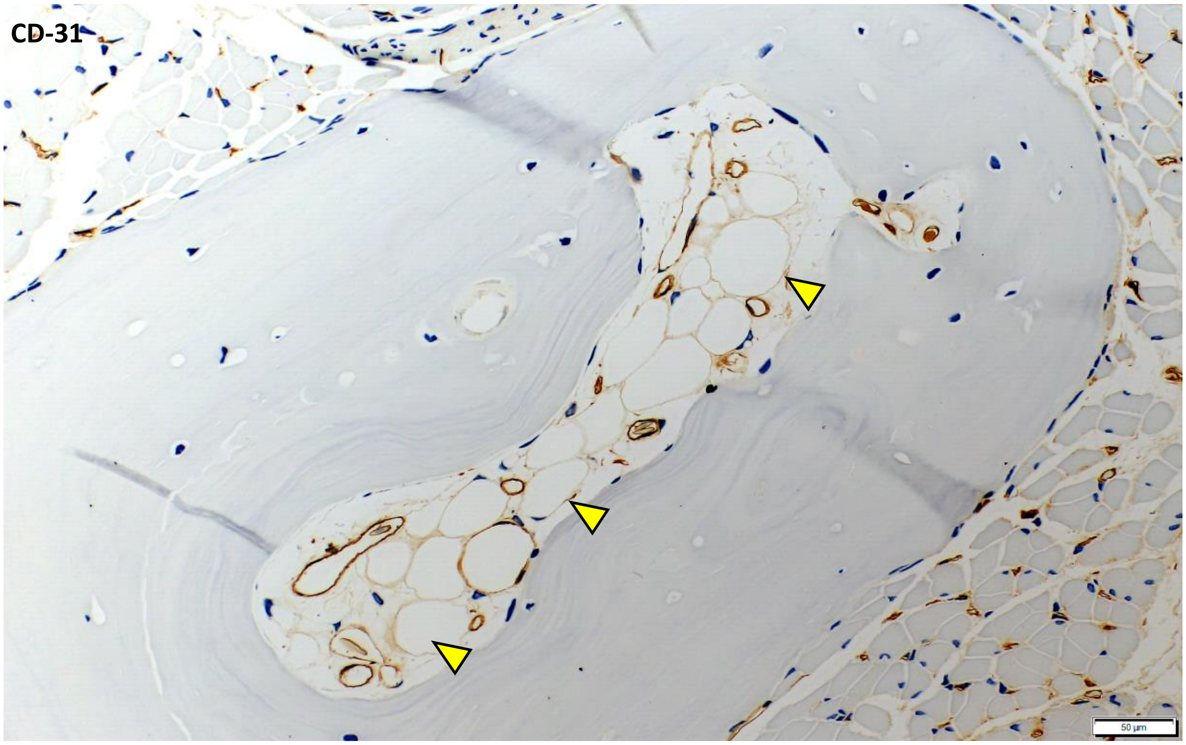

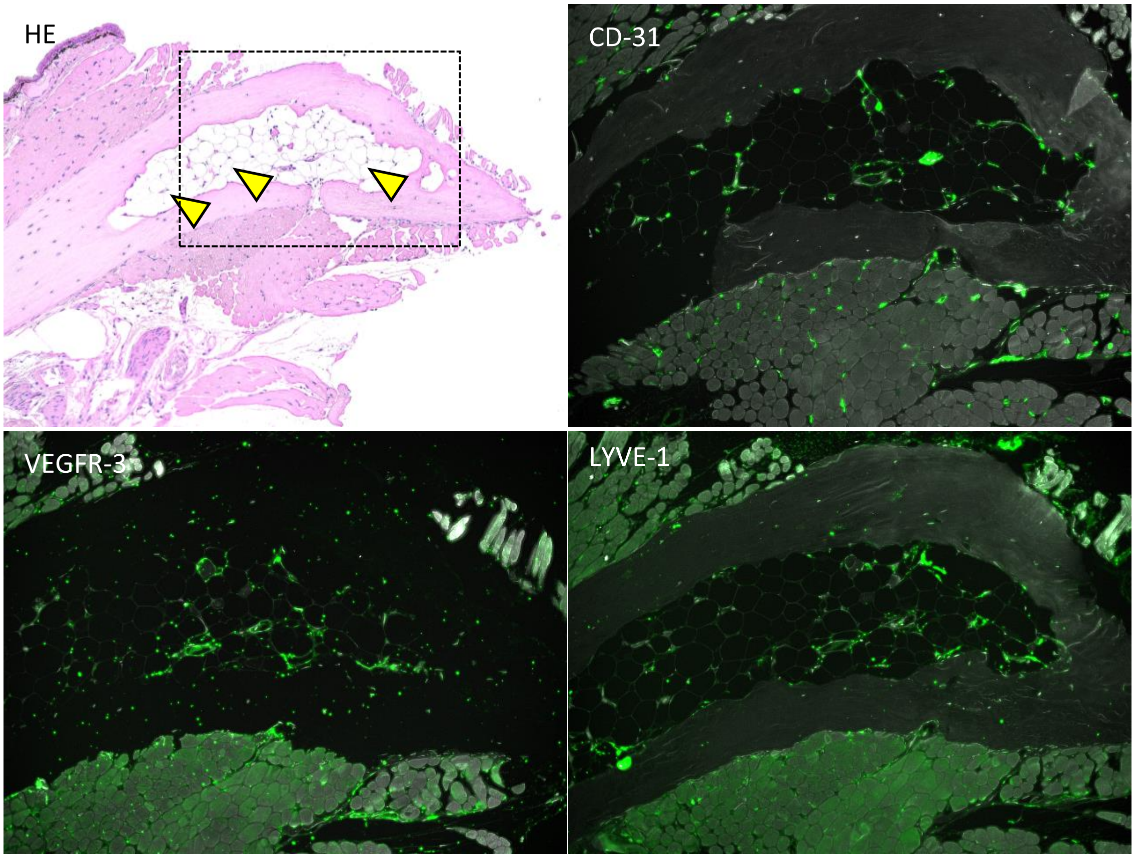

Supplementary figure (vi): Serial transverse and oblique paraffin sections 5µm thick of the newt humerus stained with HE, IHC and Immunofluorescence showing the hypocellular bone marrow filled with adipose fat tissue (yellow arrows). The bone marrow was also highly vascular with vessels expressing CD-31, VEGFR-3 and LYVE-1 found.

Vertebrae

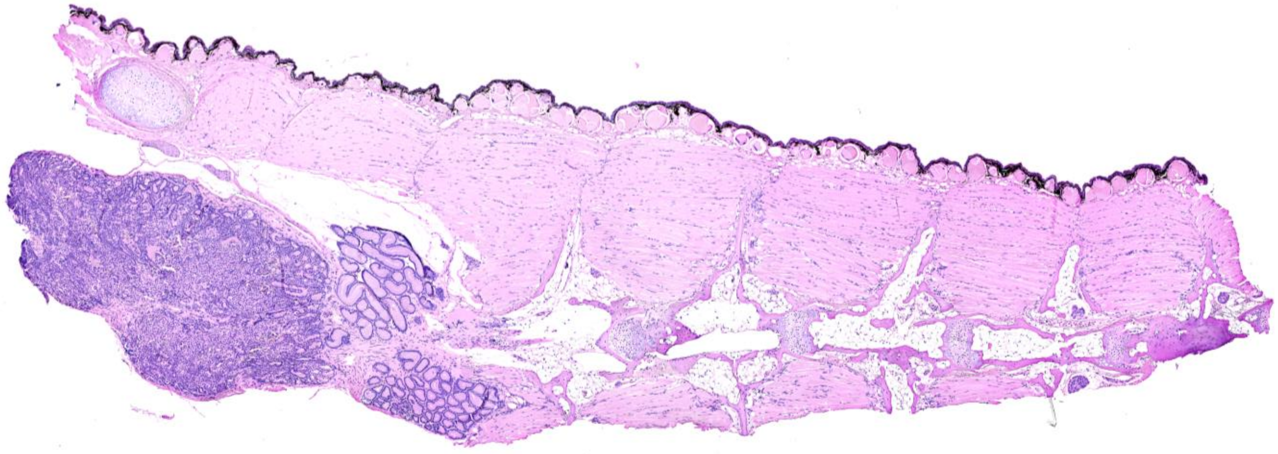

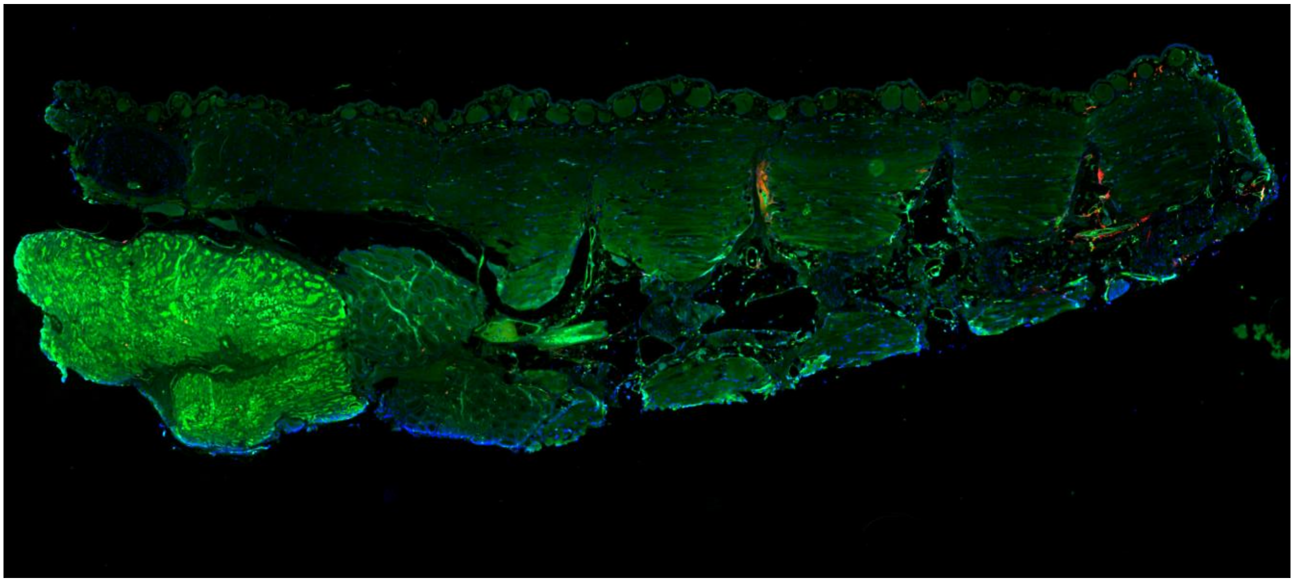

Supplementary figure (vii): Serial paraffin sections 5µm thick of the newt tail showing the hypocellular bone marrow filled with adipose fat tissue (yellow arrows). The bone marrow was also highly vascular with vessels expressing CD-31 (green), and uptake of rhodamine dextran into bone marrow lymphatics (red)

Posterior Lymphatic Heart Excision in *Xenopus laevis*

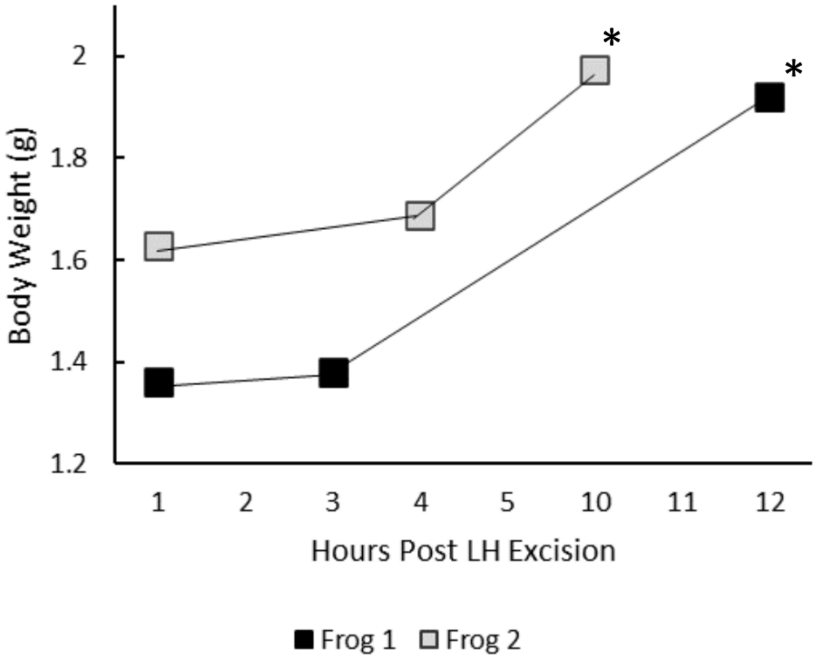

Supplementary figure (viii): Posterior Lymphatic Heart Excision Causes Edema and Death in Xenopus Laevis. After microsurgical excision of the posterior lymphatic hearts of *Xenopus laevis*, the total body weight of the frogs progressively increased, and the frogs died within 12 hours of lymphatic heart excision. This result was consistent with previous reports in anurans.[1–3] ***** Indicates death of the frog

Antibodies

Commercially Purchased Antibodies

Name	Clone	Host species	Manufacturer	Product Code	Batch Numbers	Dilution	Incubation Durations
Anti-Lyve-1[4,5]	Polyclonal	Rabbit	Novus	NB600-1008	▪ 1901R03-4<br>▪ 1901R01-3	1 : 100	▪ 1 to 3 days at 4°C<br>▪ 1 to 4 hours at room temp.
Anti-Lyve-1[6–8]	Polyclonal	Rabbit	Abcam	Ab14917	GR3392340-3	1 : 200	1 to 3 days at 4°C
Anti-CD-31[9–11]	Polyclonal	Rabbit	Bioss	BS-0195R	AH07257364	1 : 500	▪ 1 to 3 days at 4°C<br>▪ 1 to 4 hours at room temp.

Custom Made Antibody

Antibody: Rabbit Polyclonal Anti-*VEGFR-3* antibody
Manufacturer: Eurofins Genomics (Japan)
Product Code: 16445V
Position: 485-498 Based on Salamandridae (taxid:8314) VEGFR-3 orthologue
Length: 15
Sequence (N→C): NH2-C+NQNRTRKRATQRHQ-COOH
Dilution: 1 : 20 and 1 : 50
Incubation Duration: 1 to 4 hours at room temp.

References

1. Crossley, D. A. & Hillman, S. S. Posterior lymph heart function in two species of anurans: analysis based on both in vivo pressure–volume relationships by conductance manometry and ultrasound. *Journal of Experimental Biology* **213**, 3710–3716 (2010).

2. Jones, J. M., Wentzell, L. A. & Toews, D. P. Posterior lymph heart pressure and rate and lymph flow in the toad Bufo marinus in response to hydrated and dehydrated conditions. *Journal of Experimental Biology* **169**, 207–220 (1992).

3. Peyrot, S. M., Martin, B. L. & Harland, R. M. Lymph heart musculature is under distinct developmental control from lymphatic endothelium. *Dev Biol* **339**, 429–438 (2010).

4. Moon, E. H. *et al.* TMEM100 is a key factor for specification of lymphatic endothelial progenitors. *Angiogenesis* **23**, 339–355 (2020).

5. Jin, L. *et al.* Zeb1 promotes corneal neovascularization by regulation of vascular endothelial cell proliferation. *Communications Biology 2020 3:1* **3**, 1–10 (2020).

6. Wang, H. *et al.* Effect of VEGFC on lymph flow and inflammation-induced alveolar bone loss. *J Pathol* **251**, 323–335 (2020).

7. Liang, Q. *et al.* Lymphatic muscle cells contribute to dysfunction of the synovial lymphatic system in inflammatory arthritis in mice. *Arthritis Res Ther* **23**, 1–15 (2021).

8. Chi, J. *et al.* Early postnatal interactions between beige adipocytes and sympathetic neurites regulate innervation of subcutaneous fat. *Elife* **10**, 1–62 (2021).

9. Takeoka, Y. *et al.* Regeneration of esophagus using a scaffold-free biomimetic structure created with bio-three-dimensional printing. *PLoS One* **14**, e0211339 (2019).

10. Choi, B. H. *et al.* Inhibition of blood vessel formation by a chondrocyte-derived extracellular matrix. *Biomaterials* **35**, 5711–5720 (2014).

11. Zhang, H. *et al.* Dual-delivery of VEGF and PDGF by double-layered electrospun membranes for blood vessel regeneration. *Biomaterials* **34**, 2202–2212 (2013).
